# Statistical downscaling of GRACE terrestrial water storage changes based on the Australian Water Outlook model

**DOI:** 10.1038/s41598-024-60366-2

**Published:** 2024-05-02

**Authors:** Ikechukwu Kalu, Christopher E. Ndehedehe, Vagner G. Ferreira, Sreekanth Janardhanan, Matthew Currell, Mark J. Kennard

**Affiliations:** 1 School of Environment and Science, Griffith University, Nathan, QLD 4111 Australia; 2 Australian Rivers Institute, Griffith University, Nathan, QLD 4111 Australia; 3 School of Earth Sciences and Engineering, Hohai University, Nanjing, China; 4 CSIRO Land and Water, Dutton Park, QLD 4102 Australia; 5 School of Engineering and Built Environment, Griffith University, Nathan, QLD 4111 Australia

**Keywords:** Environmental sciences, Hydrology

## Abstract

The coarse spatial resolution of the Gravity Recovery and Climate Experiment (GRACE) dataset has limited its application in local water resource management and accounting. Despite efforts to improve GRACE spatial resolution, achieving high resolution downscaled grids that correspond to local hydrological behaviour and patterns is still limited. To overcome this issue, we propose a novel statistical downscaling approach to improve the spatial resolution of GRACE-terrestrial water storage changes (ΔTWS) using precipitation, evapotranspiration (ET), and runoff data from the Australian Water Outlook. These water budget components drive changes in the GRACE water column in much of the global land area. Here, the GRACE dataset is downscaled from the original resolution of 1.0° × 1.0° to 0.05° × 0.05° over a large hydro-geologic basin in northern Australia (the Cambrian Limestone Aquifer—CLA), capturing sub- grid heterogeneity in ΔTWS of the region. The downscaled results are validated using data from 12 in-situ groundwater monitoring stations and water budget estimates of the CLA’s land water storage changes from April 2002 to June 2017. The change in water storage over time (ds/dt) estimated from the water budget model was weakly correlated (r = 0.34) with the downscaled GRACE ΔTWS. The weak relationship was attributed to the possible uncertainties inherent in the ET datasets used in the water budget, particularly during the summer months. Our proposed methodology provides an opportunity to improve freshwater reporting using GRACE and enhances the feasibility of downscaling efforts for other hydrological data to strengthen local-scale applications.

## Introduction

The applications of the Gravity Recovery and Climate Experiment (GRACE) mission in hydrological modelling and assessing freshwater changes over large and meso-scale river basins have been well documented e.g.,^1–4^. Unfortunately, the coarse spatial resolution of data from the mission limits its application over smaller spatial extents, especially to support local-scale freshwater reporting and accounting. The low-spatial resolution GRACE mass concentration (mascon) solutions at 0.5° or 0.25° grids are redistributed samples of a coarser GRACE product^5^. Since these resampled grids (0.5° or 0.25°) are spatially correlated from the native resolution of 3°, they do not contain physical information at a spatial scale finer than the original GRACE resolution^6^. Consequently, for finer spatial scale GRACE estimates to be employed in effective catchment-scale hydrological assessments, improved downscaling by assimilating localized hydrological information at a higher resolution^7–9^ is critical. This study aims at introducing physical information from high resolution hydrological fluxes to improve downscaled products such that they mimic the local-scale hydrologic behaviour of a large hydro-geologic basin in northern Australia (the Cambrian Limestone Aquifer—CLA).

Machine learning regression methods are capable of effectively solving non-linear problems and have become increasingly popular in statistical downscaling operations. This is evident in recent studies that have explored regression techniques to downscale GRACE data e.g.,^10,11^. For example, Vishwakarma, et al.^6^ improved the spatial resolution of global GRACE-TWS by exploiting the dominant common statistical modes between precipitation, evapotranspiration and runoff using partial least squares regression. The downscaled products were validated by checking the conservation of mass at a catchment scale. Ning et al.^12^ developed and tested an integrated downscaling-validation procedure for GRACE derived TWS at 0.25° scale over Yunnan province, China, using a multi-linear regression method. They evaluated their downscaled product using in-situ groundwater levels, following the assumption that groundwater variations accounts for most of the TWS signals over the study region. Yin et al.^13^ designed a statistical downscaling model that uses discrete evapotranspiration data to downscale GRACE TWS based on the correlative relation method, with a proviso that the method is only feasible for regions where groundwater level variation is strongly correlated with evapotranspiration. Miro and Famiglietti^9^ implemented the artificial neural network model to predict changes in GRACE data using high resolution datasets of precipitation, temperature, soil type and slope. Their results showed that the neural network model was effective in their downscaling process but maintained the need for better estimates and finer details of the predictors variables. Other studies have used land surface models and hydrological variables based on machine learning regression methods to provide downscaled TWS estimates e.g.,^10,14–18^.

One of the most popular methods of validating the performance of downscaled GRACE-TWS is through in-situ groundwater monitoring data^19^. This is plausible because, for most regions, groundwater resources comprise over 60% of freshwater use e.g.,^13,20^ and thus contributes significantly to the changes occurring in the TWS vertical column. However, due to the challenges of the typically sparse distribution of groundwater monitoring points in space and time and other inherent data gaps (e.g., comprehensive characterisation of aquifer storage coefficients and missing observations), monitored groundwater level variations in some regions may not be suitable for quantifying water storage changes^12^. Also, while groundwater monitoring networks are essential to understand changes in aquifer storage and water budgets, we very rarely have sufficient information to quantify storage volume changes using monitoring data alone. Converting groundwater level changes to storage volume changes requires knowledge of (i) the full spatial distribution of water level changes over the relevant scales, which requires a detailed and extensive monitoring bore network and (ii) the aquifer storage coefficient (specific storage and/or specific yield). This coefficient is very rarely known across the full extent of a basin/aquifer—even at the level of general averages. Surface water and soil moisture also significantly drive the TWS changes of several regions^21^, making in-situ groundwater monitoring insufficient in itself for validating TWS over those regions.

For our study, we used in-situ groundwater levels to validate our downscaled product. We believe this is justified, because the selected region’s groundwater resources maintain a substantial contribution to the regional water budget and the GRACE column and has recorded significant recharge over the past decades^22^. This is particularly true in the southern CLA, where the climate is arid and there is very little permanent surface water. Also, since the region is well monitored, the storage coefficients/specific yield values were available and obtained from Knapton et al.^23^. We also compared the water storage changes of our downscaled product with trends from the water budget equation (ds/dt) estimated using high resolution hydrological fluxes from the Australian Water Outlook AWO^24^. We argue that the water budget estimates quantified from the fluxes of precipitation, evapotranspiration (plant transpiration and soil and canopy water evaporation) and runoff (surface and base flows) are representative of the water storage dynamics of the region. Sheffield et al.^25^ reported a reasonable similarity between global hydrological models (e.g., NOAH-VIC) model and the GRACE-TWS which depicts that hydrological model parameters can potentially close the water budget and as such we investigate whether this approach is feasible and robust for the selected case study region.

The overarching aim of this study is to downscale GRACE-TWS from April 2002 to June 2017 using high resolution hydrological model parameters of the Australian Water Outlook (AWO). The selected case study is the Cambrian Limestone Aquifer, one of northern Australia’s most important aquifer systems. Specific objectives are, (i) statistical downscaling of GRACE-TWS to 0.05° grids using precipitation, ET and runoff estimates from the AWO model, (ii) validating the downscaled TWS data using in-situ groundwater level estimates and the water budget model (ds/dt) derived from the AWO hydrological flux variables (iii) exploring the efficiency of the support vector machine (SVM) regression in establishing a functional regression model between the AWO predictors and the GRACE-TWS estimates. Regional hydrological models, such as the AWO provide detailed insights into specific regions, enabling more accurate analysis of water availability, flood potential and the impacts of land use and climate change in local scale hydrological assessments than their global counterparts. These processes offer to demonstrate an approach for optimal statistical downscaling of GRACE data representing localized hydrological trends useful for studying very small regions (0.05°), and in turn contribute to improving water management and research throughout Australia and beyond.

## Datasets

### GRACE terrestrial water storage changes

TWS as quantified by GRACE is a fundamental constituent of the terrestrial water cycle and is defined as the sum of changes in surface water, snow, ice, soil moisture, canopy storage and groundwater. Besides the significant importance of GRACE-TWS in water resources, agriculture, climate, and ecosystem monitoring, it is a key quantity for quantifying land water storage dynamics. For this study, we took an ensemble mean of GRACE level 3 mascon products from the centre for space research (CSR) of the University of Texas, Jet Propulsion lab (JPL) and the Goddard Space Flight Center (GSFC)^26^. The native resolution of the product is approximately 400 km due to the orbital altitude of the GRACE satellite. We used the filtered and processed samples of the nominal GRACE datasets which was provided at a 1.0° × 1.0° grid cell for our experiment. We computed the time series of the three GRACE-TWS anomalies relative to the long-term mean between 2004 and 2009 from the GRACE mascon field. Since we are dealing with variation of TWS over time, we obtain ΔTWS(t) from the TWS anomalies, whereby the time derivative was estimated with centred finite difference as in^27^

1 $$\Delta TWS\left(t\right)=\frac{TWS\left(t+1\right)-TWS(t-1)}{2\Delta t}$$

where Δt means one month, and *t*−1, *t,* and *t* + *1* accounts for three consecutive months. All the data used for our study is summarized in Table 1. Our study period spanned from April 2002 to June 2017.

**Table 1:** Summary of the dataset and sources used for our processing.

Variable (data source)	Source	Temporal resolution	Spatial resolution
CSR TELLUS GRACE (a)	Analysis of GRACE-TWS solutions	Monthly	1.0° × 1.0°
JPL TELLUS GRACE (a)	Analysis of GRACE-TWS solutions	Monthly	1.0° × 1.0°
GSFC TELLUS GRACE (a)	Analysis of GRACE-TWS solutions	Monthly	1.0° × 1.0°
AWO Precipitation (b)	Analysis of rain gauge data	Daily and monthly total	0.05° × 0.05°
AWO Evapotranspiration (b)	Estimate of total ET from vegetation, soil and groundwater using the event-based approach^28^	Daily and monthly total	0.05° × 0.05°
AWO Runoff (b)	Analysis of stream flow observations and satellite-based evapotranspiration and soil moisture	Daily and monthly total	0.05° × 0.05°
in-situ groundwater levels (c)	Monitoring bores	Daily	–

(a)—https://podaac.jpl.nasa.gov/, (b)—https://awo.bom.gov.au/products, (c)—http://www.bom.gov.au/water/groundwater/explorer/map.shtml (b) represents the predictors.

### The Australian Water Outlook

Our high-resolution predictor dataset is the Australian Water Outlook (AWO) package, which consists of daily gridded model outputs (precipitation, evapotranspiration, and runoff) from 1911 to 2023. The AWO system incorporates a wide range of climate inputs, downscaling techniques, post processing and assimilation of near real time satellite soil moisture states as inputs to the Australian Water Resource Assessment Landscape model AWRA-L v7^24,29^, to provide a consistent set of hydrological outputs at 0.05° grids across Australia. The absolute values of the predictors were used for the water budget estimation, while their changes (dynamics) were computed for the downscaling operation. The changes (dynamics) of each predictor variable (Table 1) are based on the removal of the long term mean of 2004–2009 from each month. This removal of long-term mean is designed to convert the datasets into a ‘net change’ in each time period (rather than absolute values), for easier comparison against the TWS variations from GRACE. All the data used in this experiment spans from April 2002 to Jun 2017 and have been summarized in Table 1.

### Groundwater level data (in-situ)

**Figure 1:**
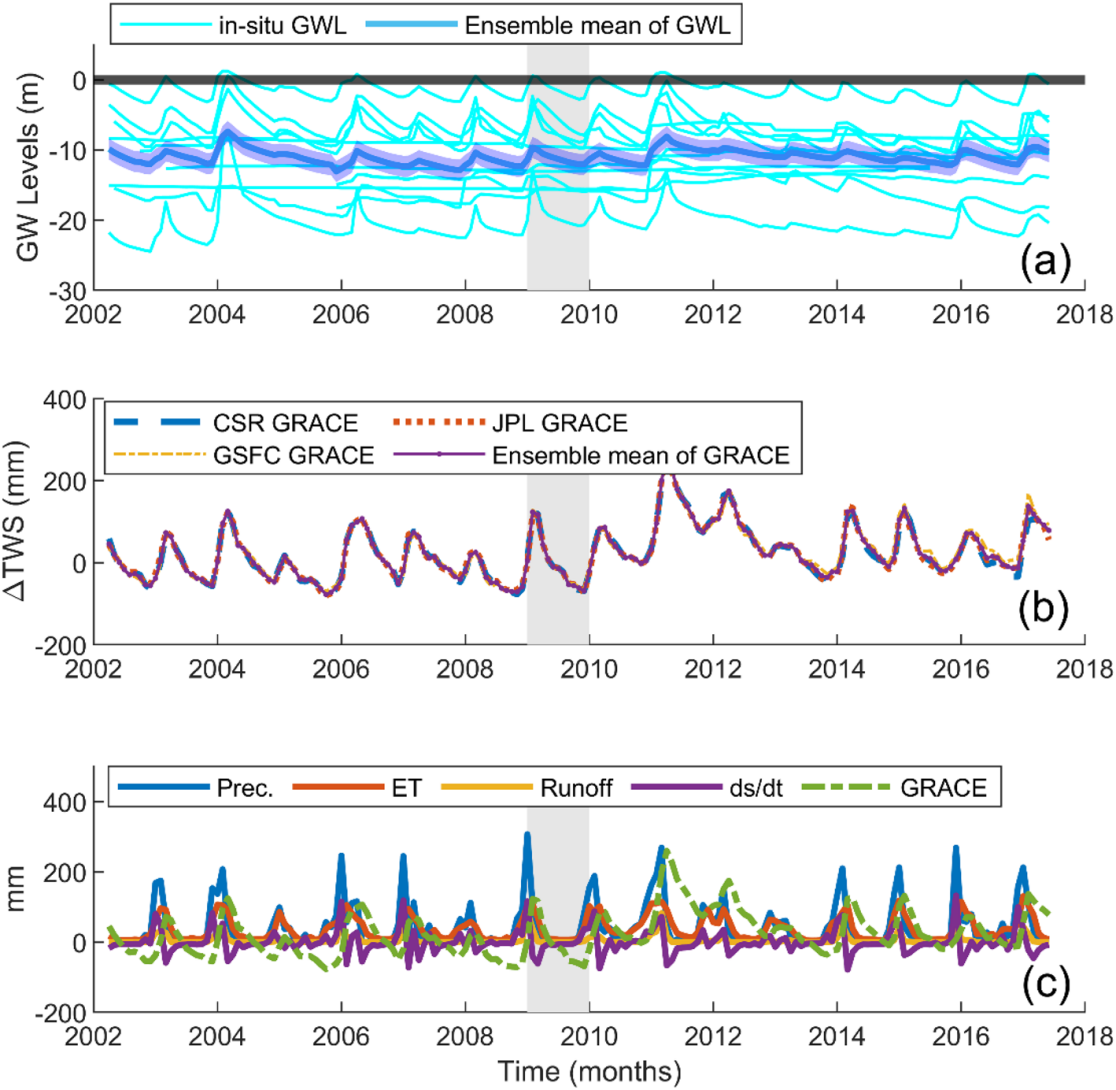
Datasets used for our experiment. (**a**) Shows the in-situ groundwater estimates after scalar multiplication from the 12 monitoring stations and their ensemble mean. The black line at the 0 y-axis represents the ground surface. Readings above the ground surface indicates artesian conditions. (**b**) Represents the GRACE products from the three processing centres, CSR, JPL and GSFC and their ensemble mean which was used for our analysis, while (**c**) shows the AWO-based hydrological flux variables of precipitation, ET and runoff and their output based on the water budget (ds/dt). The vertical grey portion shown across the entire plots depicts the end of the Australian millennium drought around 2009 and 2010. This plot was generated using MATLAB R2023a software—https://au.mathworks.com/products/matlab.html).

The groundwater level (GWL) data used in this study were compiled from the Australian groundwater explorer^30^, which provides access to a wide range of groundwater datasets, including around 900,000 bore locations and groundwater levels and is updated annually. The groundwater level term used for our experiment was the ‘depth to water (DTW)’ variable which records measurements from the top of the ground surface to the groundwater level (Fig. 1). This means that positive values are below the ground surface while negative values are above the ground surface indicating artesian conditions. Therefore, given that we are assessing below the ground surface, almost all the readings were negative. To conform the GWL time series to the other datasets (which were all positive) used in our experiment, we performed a scalar multiplication of -1 throughout the time series (Fig. 1). This operation changed the GWL time series to all positives matching the other datasets used in our experiment. Since the BOM datasets is available at daily steps, we averaged the observations from each well to months and found the ensemble mean of the GWLs from the monitoring stations. Subsequently, they were converted to ΔGWLs by removing the long term mean of 2004–2009 from each month, similar to GRACE-ΔTWS. Equation 2 shows the calculation of the ΔGWL for each month:

2 $${\Delta GWL}_{(i)}= {GWL}_{(i)}-mean GWL (2004:2009)$$

where subscript *i* represents the time in months from April, 2002 to June, 2017.

The GWL data used in our experiment was initially processed and filtered using the following criteria;

1. Bores with more than 24 months missing data were eliminated.
2. Bores whose data quality flags were rated A were retained for analysis, whereas bores rated B to F were eliminated. The data quality flag captures the quality of the data based on the supplier, with those rated A considered to be the ‘best available given the technologies, techniques and monitoring objectives at the time of classification’ (see supporting information 1c for more details on quality ratings.

The aquifer where these monitored bores are located ranges between a thickness of 100–300 m and can be described as semi-confined. Further details on the geology and classification of the aquifer levels can be found in^31^. The 12 monitoring bores used for the validation procedure are all rated quality A (supplementary information 1c). Besides bores RN008221, RN010167, and RN029429 (Table 2), every other bore had missing months, which were estimated using linear interpolation. The linear interpolation method was used due to its ease in application and prevalent utilization within the hydrologic community, however, it is important to note that this approach may induce the associated uncertainties of the in-situ groundwater storage estimates considering the non-linearity of each individual bore readings. The overall uncertainty assessment of the monitoring bores shown in Table 2 provides context to their quality and efficiency in serving as a validation tool for the downscaled GRACE product.

**Table 2:** Properties of the 12 monitoring groundwater level stations used for validation.

Bore ID	Latitude	Longitude	Bore depth (m)	Geology	Classification
RN005248	− 19.783995	134.184916	68.9	Quaternary sediments	Fractured rock
RN010167	− 19.803048	134.060717	44.0	Tertiary sediments	Fractured rock
RN010564	− 19.848769	134.158438	24.8	Tertiary sediments	Fractured rock
RN033033	− 14.133654	131.395023	90.9	Quaternary sediments	Upper aquifer
RN002522	− 14.46747	132.309845	46.9	Tindall Limestone	Lower aquifer
RN034595	− 14.588332	132.034301	37.0	Quaternary sediments	Upper aquifer
RN022394	− 14.503928	132.295794	123.6	Quaternary sediments	Lower aquifer
RN034364	− 14.069437	131.250134	37.5	Tertiary sediments	Upper aquifer
RN029429	− 14.532308	132.359108	118.0	Quaternary sediments	Lower aquifer
RN034597	− 14.598042	132.044391	24.9	Tertiary sediments	Fractured rock
RN008221	− 14.587929	132.46868	61.0	Georgina sediments	Lower aquifer
RN034596	− 14.598015	132.0444	42.8	Tertiary sediments	Fractured rock

Bore depth represents the mid-point of the screened interval, Fractured rock represents areas of hard rock between sedimentary basins.

## Case study

The region of interest is an extensive carbonate aquifer—the Cambrian Limestone Aquifer (CLA) underlying a large portion of Australia’s Northern Territory, to the north of Alice Springs and south of Katherine^32^; Supporting Information 1a. The CLA comprises three geological sub-basins; Daly, Wiso and Georgina, within which groundwater flows are inter-connected. The CLA was selected as a suitable test location for our downscaling operation because, due to the significant gradient in its climate parameters (rainfall and ET) from south to north, it may be difficult to capture the variations across the CLA with the original GRACE products, thus justifying the need for effective downscaling. The region encapsulates the entirety of the components in the GRACE vertical column (i.e., soil moisture, surface, and groundwater)^33^, and accounts for all the mass variations that GRACE captures, thus making it an ideal location for our exercise. The CLA is well-known for its abundant surface and groundwater resources which sustain the ecological and (particularly indigenous) cultural values of the region^34,35^.

The CLA’s recharge is regulated by climate and local geology—i.e., recharge is spatially restricted to areas where Cretaceous cover rocks are thin or absent^36^. At its northern limit, near Mataranka, annual precipitation averages about 800 mm and has moderately low variability from year to year. In the south, towards the Tennant creek, an averaged 400 mm has been recorded with high variability throughout the year^37^. This translates to regions north of Daly waters (Supporting information 1b) receiving relatively frequent recharge during the wet season (i.e., November–March). This is not the case in the south, as recharge occurs periodically during periods of abnormal high precipitations see^36^. The lag between such events ranges from a few years to a few decades.

## Methodology and implementation

### Statistical downscaling based on support vector machine

Hydrological variability has a strong relationship with GRACE ΔTWS at different temporal scales and orders. Conventional statistical downscaling methods have used several regression techniques for this operation using the parameters of the water budget equation e.g.,^6^. These parameters make up the predictor datasets used to downscale GRACE TWS.

**Figure 2:**
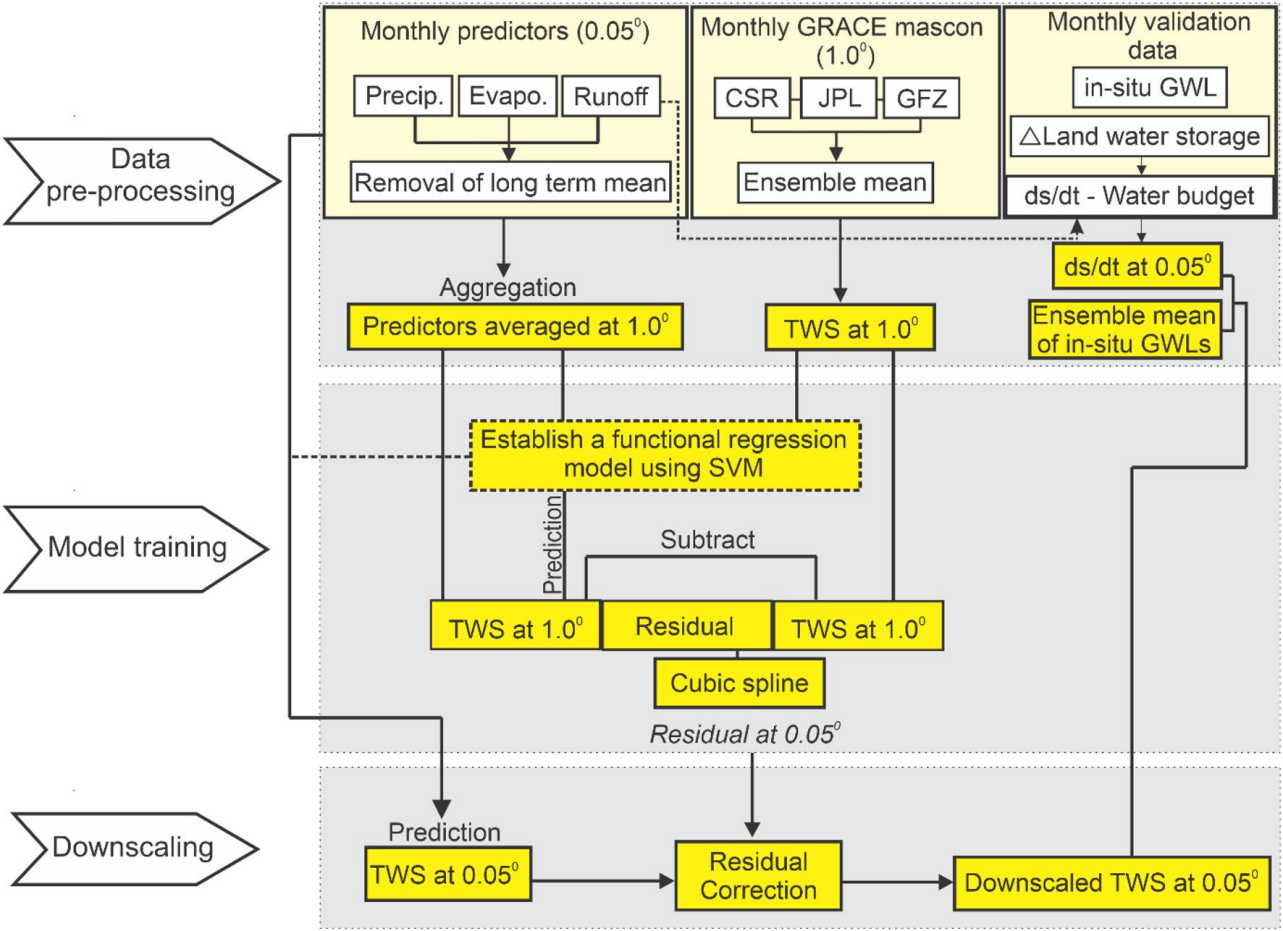
Flowchart of the downscaling process. This plot was generated using CorelDRAW v. 24.3.0.571 software—https://www.coreldraw.com/en/licensing/education/).

To achieve consistency in the spatial grain size of the predictor and predictand variables, we used pixel averaging to aggregate the independent variables (i.e., precipitation, evapotranspiration, and runoff) changes derived from AWRA-L to 1.0° × 1.0° to match the grain size of the dependent variable (GRACE-ΔTWS). An empirical functional regression model^38^ was established between the dependent and independent variables using the SVM regression (Fig. 2).

The SVM is regarded as a non-parametric technique due to its reliance on kernel functions e.g.,^39,40^. We used the polynomial kernel to map the aggregated model into a high-dimensional feature space.

3 $$f\left(x\right)= {w}^{T}\varphi \left(x\right)+b$$

where $\varphi$ represents the non-linear mapping function, and the respective weights and bias terms are represented by w and b. The SVM optimization model is given by;

4 $$\genfrac{}{}{0pt}{}{min}{w,b,\xi ,{\xi }^{*}} 0.5\times {w}^{T}w+C\sum_{i=1}^{n}({\xi }_{i}+ {{\xi }_{i}}^{*})$$

5 $$s.t\left\{\begin{array}{c}{y}_{i}-\left(\left\{w,{x}_{i}\right\}+b\right)\le \varepsilon +{\xi }_{i}\\ \left(\left\{w,{x}_{i}\right\}+b\right)-{y}_{i}\le \varepsilon +{{\xi }_{i}}^{*}(i=\mathrm{1,2},\dots ,l)\\ {\xi }_{i}, {{\xi }_{i}}^{*} \ge 0\end{array}\right.$$

*l* and n represent the number of samples, ${\xi }_{i}, {{\xi }_{i}}^{*}$ represents the upper and lower training errors, respectively, ${x}_{i}$ and ${y}_{i}$ represents the inputs and outputs of the training data, respectively, $\varepsilon$ and C represents the insensitive loss factor and the regularized constant, respectively.

To generate the prediction function, *f*($\cdot ,\cdot ,\cdot$), we use the Lagrange multipliers ${a}_{i}$ and ${{a}_{i}}^{*}$ as follows;

6 $$f\left(x,{a}_{i},{{a}_{i}}^{*}\right)= \sum_{i=1}^{n}\left({a}_{i}- {{a}_{i}}^{*}\right){K}_{P}\left(x,{x}_{i}\right)+b$$

${K}_{P}$ is the polynomial kernel function and is represented by;

7 $${K}_{P}\left(x,{x}_{i}\right)={(1+{x}^{T}{x}_{i})}^{2}$$

The superscript, 2 represents the order of the polynomial kernel used in our learning process.

Using the regression function derived from Eq. (6), we predicted GRACE-TWS and extracted the residual between the predicted and original GRACE-TWS. The residuals account for the amount of GRACE-TWS that cannot be predicted by our regression model that may reflect the influence of climate change and anthropogenic effects (e.g., water extraction) on the CLA’s land water storage interactions^32^. Since the polynomial coefficient of the residual values have an interval of 1.0° × 1.0° grids, we applied cubic spline interpolation to make it consistent with the predictor spatial resolution of 0.05°. Cubic splines are continuous curves that involve fitting a series of cubic polynomials to the data in a way that ensures smoothness. It has the advantage of preserving the information contained in the original dataset and often provides higher-order accuracy than linear or lower-degree polynomial interpolation. It can be designed to have ‘natural’ boundary conditions, where the second derivatives at the endpoints are set to zero and this enables a more stable and well-behaved interpolation. This tends to produce a more accurate representation of the underlying function, especially when the data points are closely spaced. This was implemented in our residual value by fitting the low 1.0° × 1.0°—degree polynomials to five subsets of values obtained by subtracting the lower endpoint of corresponding knot intervals in a conventional polynomial equation as in Eq. (8).

8 $$f\left(x\right)={a(x- {x}_{1})}^{3}+{b(x- {x}_{1})}^{2}+c\left(x- {x}_{1}\right)+d$$

a, b, c, and d represents the coefficients on the interval [*x*, *x*_1_].

The regularized constant represented as C in Eq. 4 uncovers the trade-off between the flatness of the function and the amount up to which the differences larger than $e$ are permitted when it is greater than 0^41^. This is similar to the process of handling a so-called $\varepsilon$-insensitive loss function ${|\xi |}_{\varepsilon }$ described in Smola and Schölkopf^42^ as

9 $${|\xi |}_{\varepsilon }= \left\{\begin{array}{l}0\quad if\; \left|\xi \right| \le \varepsilon \\ \left|\xi \right|-\varepsilon \quad otherwise\end{array}\right.$$

We favoured the polynomial kernel in this operation because it represents the similarity of training samples in a feature space over polynomials of the original variables, which improves the learning of non-linear climatic models as has been reported in past literatures e.g.,^39^. The strength of our machine learning procedure is determined by the magnitude of the residuals (Supporting information 2). It is possible that other robust machine learning models could provide lower residuals than the SVM in this scenario, however, this can be explored in future research.

After GRACE-TWS was predicted using the regression model in Eq. 6, the final downscaled GRACE-TWS was obtained by adding the interpolated residuals back to the predicted GRACE-TWS. Our entire downscaling approach is represented in Fig. 2.

### Validation and water budget compatibility assessment

To validate our downscaled product, we used in-situ groundwater levels from the Australian Groundwater Explorer consisting of 12 monitoring bores, unevenly spread across our study region (Table 2). We also assessed the water budget fit on the downscaled products using AWO’s high resolution variables, i.e., precipitation, evapotranspiration, and runoff. This was to test if the water budget is maintained by the downscaled product The water budget equation (Eq. 10) illustrates the water interchange between the ocean, land, and atmosphere. It provides a unique representation of land water storage changes based on hydrologic fluxes and has been shown to maintain a significant and similar trend to what GRACE measures e.g.,^25,27^.

10 $$|P-ET| =|R|+|\frac{ds}{dt}|$$

Equation 10 expresses the sum of water gained by a catchment in the form of precipitation (P), as the total amount of water returning to the atmosphere through evapotranspiration (ET), water leaving the basin through runoff (R), and any variations in the basin’s terrestrial water storage (expressed as ds/dt). The state variables P, ET, R and ds are areal averages of distributed absolute values so that the sign | | indicate spatial averaging over the entire basin throughout the duration of our study period. We also explored the use of other statistical approaches in our validation as discussed in what follows.

### Statistical rotation

We used the principal component analysis (PCA) technique to evaluate the spatio-temporal consistency between the original and downscaled GRACE-TWS. PCA is a dimension reduction technique that is well known for its efficiency in minimizing the dimensionality of large multivariate data^43–45^ while accounting for the strongest dominant variations in the data^46^. Determining the spatio-temporal consistency between the original and downscaled TWS estimates is very important to assess the similarity of both original and downscaled product, and this can be achieved by maintaining a significant correlation between the PC’s of the two datasets. The correlation signifies that some, most or all the information contained in one variable (original TWS) is also contained in the other variable (downscaled TWS)^47^. Also, the PCA’s ability to isolate long-term signals and inter-annual periodic variations warrants its use in this context e.g.,^48^.

11 $$\left[\begin{array}{c}{y,\widehat{y}}_{T,1}={L}_{11}{x,\widehat{x}}_{T,1}+{L}_{12}x,{\widehat{x}}_{T,2}+{L}_{13}x,{\widehat{x}}_{T,3}+\dots +{L}_{1K}{x,\widehat{x}}_{T,K}\\ {y,\widehat{y}}_{T,2}={L}_{21}{x,\widehat{x}}_{T,1}+{L}_{22}x,{\widehat{x}}_{T,2}+{L}_{23}x,{\widehat{x}}_{T,3}+\dots +{L}_{2K}x,{\widehat{x}}_{T,K}\\ \dots \\ \dots \\ {y,\widehat{y}}_{T,K}={L}_{K1}x,{\widehat{x}}_{T,1}+{L}_{K2}{x,\widehat{x}}_{T,2}+{L}_{K3}x,{\widehat{x}}_{T,3}+\dots +{L}_{KK}{x,\widehat{x}}_{T,K}\end{array}\right] T=1,\dots ,183$$

The original and downscaled matrix $x$ and $\widehat{x}$ contains rows depicting the time T in months and K, the variables. L represents the loadings which provides the weights of the original variables in the principal components (PC). The $y$ and $\widehat{y}$ values represent the orthogonal original and downscaled PCs, with ${y,\widehat{y}}_{T,1}$ explaining the highest variability and ${y,\widehat{y}}_{T,2}$ to ${y,\widehat{y}}_{T,K}$ representing the remaining variance. For our validation exercise, we restricted the PCs to ${y,\widehat{y}}_{T,1}$ and ${y,\widehat{y}}_{T,2}$. The first PC is the linear combination of the original parameters that contributes the largest to the total variance; the second PC, uncorrelated with the first one, contributes the largest to the residual variance, this process continues until the total variance is analysed. Since the method is so dependent on the total variance of the original variables, we decided to normalize the variables. Hence, our final PCs were unitless.

We analysed the spatial patterns of the original and downscaled products using the eigenvectors, which is also referred to as the empirical orthogonal functions (EOFs). The EOFs which represent the spatial distribution of the original and downscaled products over time were generated from the sample covariance matrix of the centred data matrix for x and $\widehat{x}$, respectively.

### Estimating in-situ based groundwater storage anomalies (GWSA)

The groundwater levels were converted to storages based on the storage coefficients and specific yields of the CLA’s karstic aquifer^49,50^:

12 $${GWSA}_{in-situ}=({h}_{m}\times A\times {S}_{y\left(c\right)}- {h}_{i}\times A\times {S}_{y\left(c\right)})$$

where ${h}_{m}$ and ${h}_{i}$ represents the long-term mean of the GWL and GWL depths at different time periods, respectively, A is the area influenced by the bores (in this case, the entire CLA) and ${S}_{y\left(c\right)}$ represent the specific yield/storage coefficient of the CLA. The CLA is a karstic aquifer majorly composed of limestone. It is overlain and confined by shale, sandstone, and dolostone from the Ordovician siltstone. The karstic nature of the aquifer mean that its formation exhibits very high transmissivities (> 5000 m^2^/d for the Cambrian limestone) and relatively low specific yield/storage coefficient with estimates ranging from 0.01 to 0.06^23^.

### Seasonal trend and variability index

To further validate the downscaled products, we explored its consistency with in-situ GWS changes over different seasons with varying hydrological conditions. The north of Australia (where the CLA is located) has a pronounced dry (autumn–winter) and a wet (late spring–summer) season. However, to capture most of the seasonal changes, we split them into Austral summer, autumn, winter, and spring seasons ranging from December to February, March to May, June to August, and September to November, respectively.

To estimate the seasonal trends for each grid in the original and downscaled products, we utilized a seasonal partitioning technique:

13 $${\Delta TWS}_{1.0, 0.05}=\left[\begin{array}{ccccc}{\Delta }_{\mathrm{1,1}}& {\Delta }_{\mathrm{1,2}}& {\Delta }_{\mathrm{1,3}}& \dots & {\Delta }_{1,n}\\ {\Delta }_{\mathrm{2,1}}& {\Delta }_{\mathrm{2,2}}& {\Delta }_{\mathrm{2,3}}& \dots & {\Delta }_{2,n}\\ {\Delta }_{\mathrm{3,1}}& {\Delta }_{\mathrm{3,2}}& {\Delta }_{\mathrm{3,3}}& \dots & {\Delta }_{3,n}\\ \vdots & \vdots & \vdots & \dots & \vdots \\ {\Delta }_{\mathrm{183,1}}& {\Delta }_{\mathrm{183,2}}& {\Delta }_{\mathrm{183,3}}& \dots & {\Delta }_{183,n}\end{array}\right]$$

where n represents the number of grids over the CLA. The value of n for the 1.0° and 0.05° grids are 169 and 68,121 respectively. Row 1–183 represents April 2002 to June 2017 in months. Therefore, one seasonal cycle which is from Jan to Dec has 12 months. Equation 10 was partitioned into different seasons by applying,

14 $$season(i)= \Delta TWS\left[a:b\right] :12 :[183]$$

a and b signify the months for the respective seasons. For example, 12:2 depicts summer, 3:5 depicts autumn, 6:8 depicts winter and 9:11 depicts spring. This operation was performed for the downscaled GRACE against the original GRACE, ΔGWSs and ds/dt estimates.

To test for parametric trends on the downscaled and in-situ ΔGWS products, we employed the Mann–Kendall test^51–54^.

15 $$MK= \sum_{k=1}^{n-1}\sum_{j=k+1}^{n}sgn \left({\Delta TWS}_{j}- {\Delta GWL}_{i}\right) \mathrm\;{for }\;1 \le i<j\le n$$

where MK denotes the Mann–Kendall statistic, n is the time in months over the study region, ${\Delta TWS}_{j}\,and\,{\Delta GWL}_{i}$ represents the data values at time $j and i (j>i)$.

The MK test statistic represents the positive and negative transformation for all significant grid points^3^. Under the null hypothesis, the statistics mean (E[M]) = 0, and the variance (σ) is depicted as;

16 $$\sigma =\frac{n\left(n-1\right)\left(2n+5\right)- \sum_{k=1}^{m}({t}_{k}-1)({2t}_{k}+5)}{18}$$

where n is the number of data points, m is the number of sample datasets having the same value and ${t}_{k}$ is the number groups of data points that have k identical values. In our case where the sample size is 177 (complete yearly cycles running from January to December) we computed the standard normal test statistic (${Z}_{t}$) based on the Z-transformation given below,

17 $${Z}_{t}=\left\{\begin{array}{c}\frac{MK-1}{{[\sigma]^\frac{1}{2}}} \;if\; MK>0\\ 0,\; if \;MK=0\\ \frac{MK+1}{{[\sigma]^\frac{1}{2}}},\; if \;MK<0\end{array}\right.$$

This test is estimated to be Gaussian. The null hypothesis (H_0_) which indicates no trend was tested at a 95% confidence level.

### Model performance evaluation

To evaluate the performance of our downscaled product, we applied the root mean square error (RMSE), Nash–Sutcliffe efficient coefficient (NSE) and mean absolute error (MAE). These statistical tools have been extensively applied in the performance evaluation of several hydrological models^55,56^ and are given by;

18 $$RMSE=\sqrt{\frac{1}{n}\sum_{i=1}^{n}{({\Delta TWS}_{i}^{(0.05) }-{\Delta GWS}_{i})}^{2}}$$

19 $$NSE=1-\frac{{\sum }_{i=1}^{n}{({\Delta TWS}_{i}^{(0.05) }- {\Delta GWS}_{i})}^{2}}{{\sum }_{i=1}^{n}{({\Delta TWS}_{i}^{(0.05) }-{\Delta GWS}_{i} )}^{2}}$$

20 $$MAE=\frac{1}{n}{\sum }_{i=1}^{n}\left|{\Delta TWS}_{i}^{(0.05) }- {\Delta GWS}_{i}\right|$$

where n in Eqs. 18–20 represents the total number of estimates in months, ${\Delta TWS}_{i}^{(0.05)}$ and ${\Delta GWS}_{i}$ represents the downscaled GRACE and in-situ groundwater storage changes, respectively.

## Results and discussion

### Technical capability of our downscaling process and trend test

We demonstrate the capability of the SVM regression in downscaling GRACE ΔTWS signals from 1.0° to 0.05°. For the grid based SVM regression approach, our goal was to find a function f(x) that had the most deviation from the actually obtained targets for all the training data, and at the same time is as flat as possible. This means that we are not concerned about errors as long as they are less than $\varepsilon$ for each grid. Since our aim was to establish a functional regression relationship between the high-resolution predictors and the GRACE-TWS, we were looking for a function that approximates all pairs of $\left({\Delta P,ET,R}_{AWO}, {\Delta TWS}_{GRACE}\right)$ with $\varepsilon$ precision or in other words, an *f*(*x*) whose convex optimization function is feasible (Eq. 5). Since this was difficult to achieve after minimizing f(x), we increased the threshold for the error margin and introduced some slack errors ${\xi }_{i}, {\xi }_{i}^{*}$ (Eq. 4) to cope with the infeasible constraints of the optimization problem^42^. This same idea was used by^57^ to introduce a soft margin loss function which was later used in the support vector machines by^58^.

**Figure 3:**
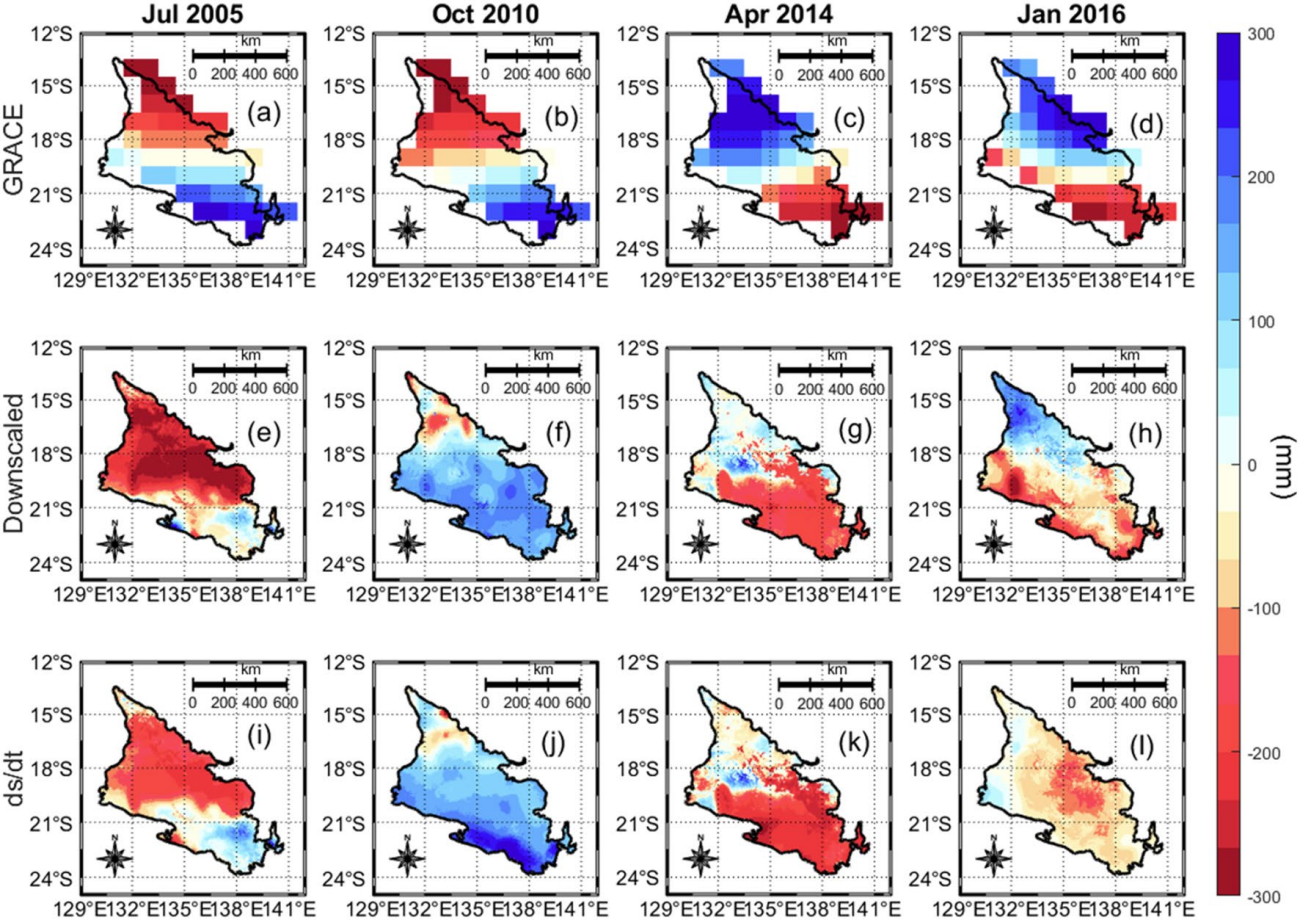
Terrestrial water storage changes over the CLA during the peak Austral winter (July), spring (October), autumn (April) and summer (January) seasons for selected years. The selected slices of July, October, April, and January are aimed at representing the mid-months for the winter, spring, autumn, and summer seasons, respectively. The first row contains an ensemble of the three CSR, JPL and GSFC GRACE mascon products, the second row contains the downscaled product based on the SVM-regression, and the last row shows the storage dynamics of the catchment based on the water budget changes (ds/dt) for the specific epochs shown in the columns. The individual downscaling of the CSR, JPL and GSFC products are shown in Supporting information 3, 4, and 5, respectively. This plot was generated using MATLAB R2023a software—https://au.mathworks.com/products/matlab.html).

After the regression model was established, GRACE ΔTWS was predicted at 1.0° × 1.0°. The predicted samples were subtracted from the original samples to highlight the residuals. These residuals account for complex signals that the SVM model was unable to capture. The established regression model was then used to predict GRACE ΔTWS at 0.05° × 0.05°. After the prediction, it was important to add back the residual to the initial prediction through the residual correction process. Residual correction is vital because it fine tunes the downscaled product by adjusting for unmodelled fine-scale changes, thereby making sure that the downscaled estimates not only depict fine-scale details, but also improves the representation of regional and local conditions accurately^56^. The result of the SVM downscaling operation is shown in Fig. 3 for the peak Austral winter (July), spring (October), autumn (April), and summer (January) seasons for years 2005, 2010, 2014, and 2016, respectively.

After the downscaling process, it was important to assess the trends between the original and downscaled products. This was particularly pertinent to detect significant trend variations that might have occurred with the downscaled products tracking finer scale changes in water storage, especially in a hydrological complex region like the CLA. In Table 3, the Mann Kendall trend test result for the original and downscaled product is shown.

**Table 3:** Man–Kendall trend test result at alpha = 0.05 to find the trends for the original GRACE, downscaled GRACE, and in-situ ΔGWS.

Test	Original GRACE	Downscaled GRACE	In-situ ΔGWS
	H = 1	H = 1	H = 0
*P*-value	0.0003	0.0002	0.5318
Trends	0.3782	0.3505	− 0.0192

When H = 1, we reject the null hypothesis which means that a monotonic trend is present and when H = 0, we assume that there is insufficient evidence to reject the null hypothesis which means that no monotonic trend is present.

The test was performed for the entire study period i.e., April 2002–June 2017.

Table 3 shows positive trends for both the original and downscaled GRACE but showed negative trends for the in-situ ΔGWS values. This trend results reveal that, while the downscaled product provides information of finer-scale details, the new information based on the CLA’s hydrological dynamics was not enough to change its trend from the original GRACE data. For the in-situ ΔGWS, no monotonic trend was observed. This clearly shows a balance in the water budget of the CLA represented by corresponding recharge and discharge of groundwater in the region.

### Testing the water budget fit on the downscaled product

The water budget was tested to see how well they fit the downscaled product. We estimated the water budget equation by improving the quantification approach of the CLA’s water storage dynamics^59,60^. This approach helped in minimizing uncertainty in our water budget estimation.

The water budget process in Eq. 10 is a universal concept used to explain the land water storage dynamics experienced in any catchment. This equation obeys the principle of conservation of mass and has been shown to be an indispensable tool for validating our understanding of catchment-water cycle^6,25,27^. One of the complications in the application of the equation in the context of GRACE data is the potential mismatch between the boundaries of surface and groundwater catchments, and the potential significant lag-times in the response of large groundwater systems to changes in other hydrological variables in the equation. The first of these issues is overcome in the current study by taking the extent of the CLA groundwater basin in its entirety (which contains numerous surface water sub-catchments), as the area of study.

**Figure 4:**
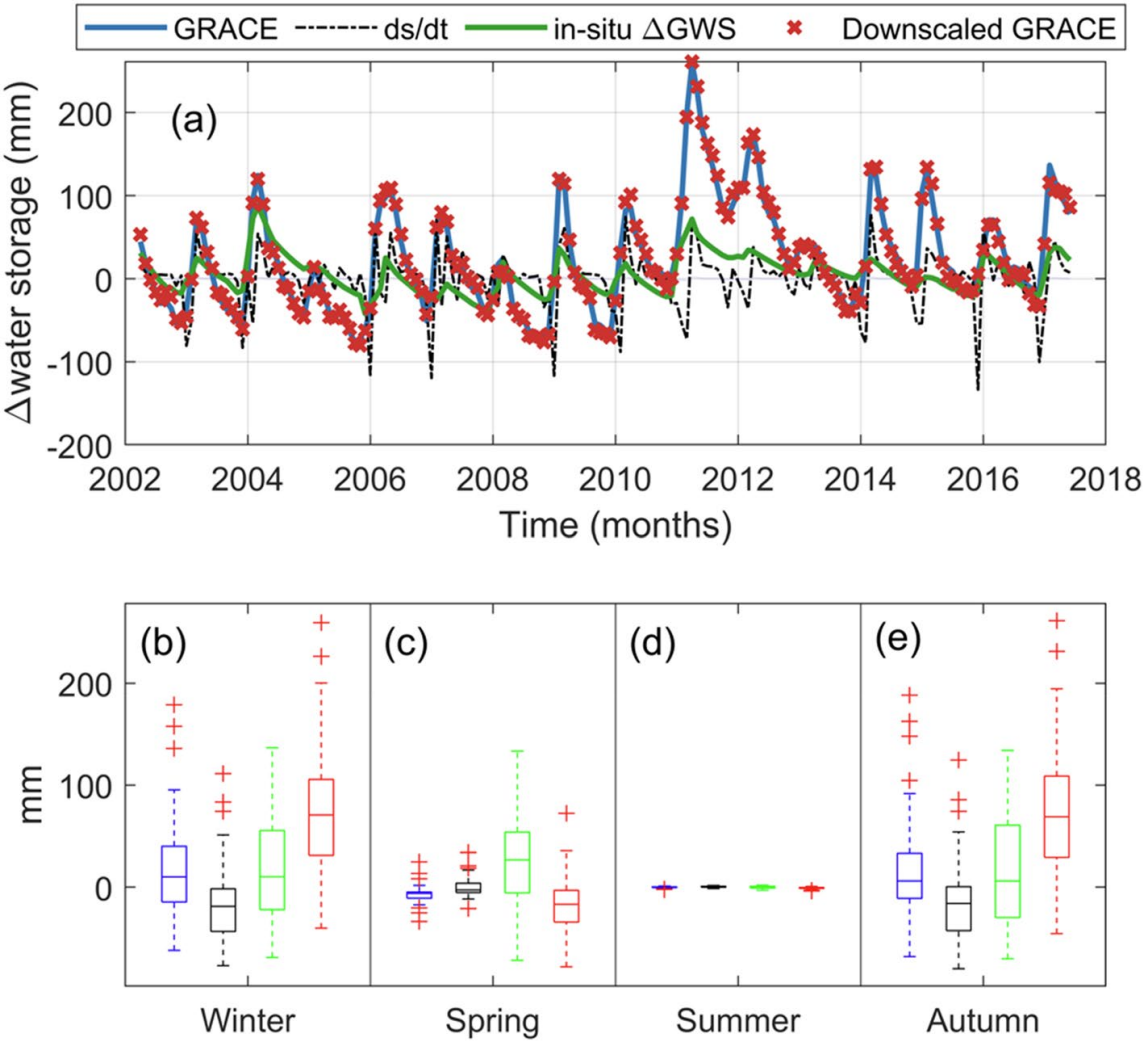
**a** Time series of downscaled GRACE against the water budget trends (ds/dt) and observed in-situ groundwater level changes. (**b**–**d**) provides a visualization of the summary statistics for GRACE, water budget (ds/dt), in-situ ΔGWS, and downscaled GRACE, which are represented by the blue, black, green and red box plots, respectively, over the four seasons. The top and bottom of each box are the 25th and 75th percentiles of the observations, respectively. The distance between the bottom and top of each box is the interquartile range. Observations beyond the whisker length (i.e., lines extending above and below each box) are outliers and indicated with + symbols. This plot was generated using MATLAB R2023a software—https://au.mathworks.com/products/matlab.html).

For our study period and region, GRACE-TWS depicted a steady inter-annual trend while the water budget was able to capture intra-annual variations (Fig. 1c). This shows the robustness of regional hydrological models in monitoring relatively smaller, rapidly responsive catchments. This is important in downscaling because, the hydro-climatic actions, like climate oscillations and anthropogenic forcings that drive the multi-annual trends of regional models over small catchments are introduced as additional information in our downscaled product. Another interesting feature in the temporal patterns of the water budget ΔTWS and the GRACE-ΔTWS is the time lag. Knowledge of time lag is important for understanding the longest period over which the available stored freshwater resources can be sustainably exploited after the rainy seasons. The peak amplitude of the water budget (ds/dt) was between December and February while the peak amplitude for the downscaled GRACE-ΔTWS was from February to April throughout our study period (Fig. 4).

Xu et al.^61^ pointed out that when precipitation is converted to TWS during the water distribution process, there exists a possibility of a theoretical delayed response between TWS and precipitation. Since ds/dt is modelled after hydrological fluxes, precipitation being the most dominant, this case holds for our study region. The delayed response of 1–2 months (Fig. 4) in GRACE-TWS observed when water enters the system as precipitation and distributes into the surface and sub-surface waters suggests that precipitation is the major driver of TWS over the CLA. Along with climatic factors, aquifer properties over the CLA such as the permeability and specific storage properties of the aquifer sediments (inter-layered limestone and mudstone)^32^ are the main driving force behind the delayed response of water budget (ds/dt) and GRACE ΔTWS^62^. For example, Awange et al.^63^ reported a 6-month delay for aquifers characterized by unconsolidated sediments and a 0-month delay in Karst dominated aquifer in Ethiopia. Similarly, the CLA is composed of karstic features (sinkholes and dolines) and fracturing underlain by older Cambrian volcanic rocks. Recharge to the CLA is thought to be somewhat restricted by the extent of overlying, younger Creteaceous rocks (mudstone, sandstone, and clay) above the CLA^36^. It may therefore be plausible to attribute the time lag in TWS observed in Fig. 4a to the aquifer’s capacity to transmit climatic variations into changes in recharge and storage.

### Validation and accuracy estimation of the downscaled product

#### Temporal and spatial variability

In the absence of other satellite based TWS product(s) or in-situ data for a direct comparison to GRACE, validating gridded downscaled ΔTWS estimates is difficult. Nevertheless, apart from the use of in-situ GWS and the water budget model, we validate the efficacy of our downscaled product by assessing the space–time consistency between the original and downscaled products. This was achieved by employing PCA technique to calculate the principal components and eigenvectors of the original and downscaled datasets. We examined the eigenvectors for both datasets and the eigenvalues associated with each principal component. The first three PCA modes which gave a cumulative variance of 96.3% and 96.9% for the original and downscaled products, respectively, were adopted as meaningful signals representing most of the total TWS variability of CLA for both scenarios.

**Figure 5:**
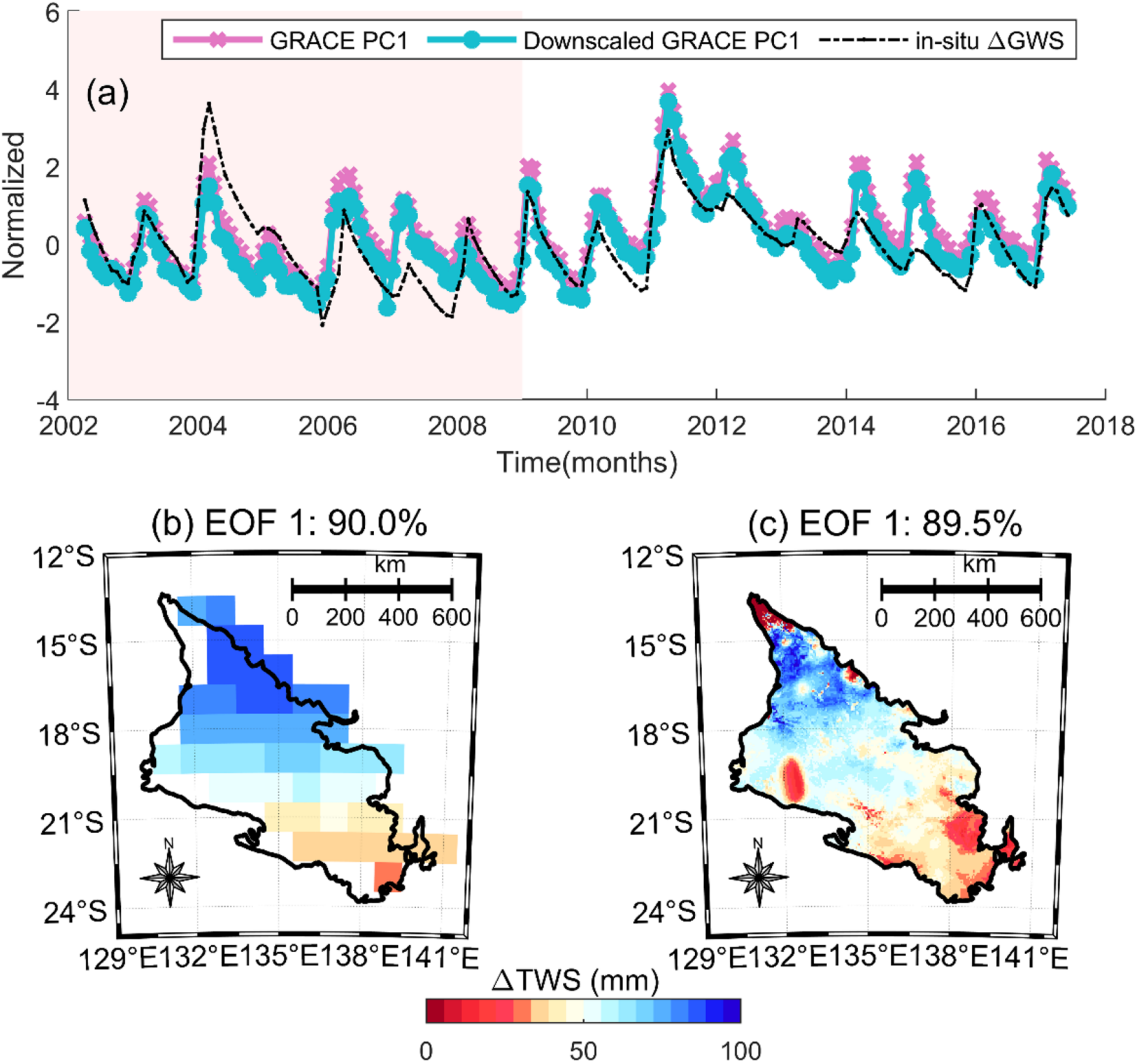
Validating our downscaled ΔTWS (PC 1) by checking its spatio-temporal consistency with the in-situ GWS changes and the original ΔTWS using principal component analysis. The pale red in (**a**) ranging from 2002 to 2009 represents the period of the Australia’s millennium drought which ended in 2009/2010. (**b**) and (**c**) depicts the empirical orthogonal functions (EOFs) of the original and downscaled GRACE, respectively. The EOFs are loadings showing spatial patterns of ΔTWS over the CLA while the corresponding PC1 (**a**) are temporal variations which are normalized using their standard deviations to be unitless. This plot was generated using MATLAB R2023a software—https://au.mathworks.com/products/matlab.html).

The first PCA mode which explains 90.0% and 89.5% of variance for the respective original and downscaled variance (Fig. 5), depicts the annual variability of TWS changes over our test bed. This mode shows that the strongest annual variability (+ ve) over the CLA is prominent over the Daly basin (northernmost section of the CLA). These strong spatial loadings in the north of the CLA are largely precipitation-driven, contributing to the relatively high annual recharge rates in the region. This is in line with the findings of Bruwer and Tickell^64^ who estimated recharge to the Daly basin CLA (Tindall Limestone) to be approximately 330 GL/year greater than the other sub-basins to the south and less variable between years, as well as point-based diffuse recharge estimation by Crosbie and Rachakonda^22^. Significant surface runoff (following wet season monsoons), groundwater recharge, and discharge to the rivers in the basin (e.g., Daly, King Roper, and Flora) which are gaining streams (receiving groundwater discharge) along most of their length are likely responsible for the variability in the Daly^32,65^. This relatively high variability over the Daly basin results is captured in their corresponding PCs showed in Fig. 5a. It is also important to note the spike in the amplitude of PC1 around 2011. This spike was as a result of the heavy rainfalls between 2009 and 2010 that coincided with the end of the Australia millennium drought which was predominant in Southern Australia^66^. This signal was also captured by the in-situ GWS at around the same period (Fig. 5a).

The second and third PCA modes (Supporting information 6 and 7) explains 7% and 2.3%, respectively, for the original GRACE and 4.5% and 3.2%, respectively for the downscaled products. We categorize them as greater intra-annual variations as they depicted more consistent inter-annual variations. However, due to their minimal variance, they maintained little relationship with the in-situ ΔGWS and may not provide a comprehensive characterization of TWS dynamics over the CLA. Most of these greater intra-annual signals are coming from eastern Wiso Basin and the North-western Georgina Basin. This variability is likely to be caused by ephemeral surface water bodies, seasonal flows and/or soil moisture in the region^32,67^. It is safe to conclude that the Daly basin witnesses more consistent variability in total water storage regardless of its relatively smaller size (Fig. 5, Supporting information 6, 7). This means that the TWS here has a stronger seasonal change compared to the other regions of the CLA and this is consistent with the much higher total rainfall and more reliable wet/dry season experienced in the northern territory. Since the variability of catchments being significant, is not hindered by their sizes (Fig. 5c); it portrays the usefulness of downscaling the GRACE ΔTWS estimates to effectively monitor hydrological operations in relatively small scales.

#### Seasonal variability

**Figure 6:**
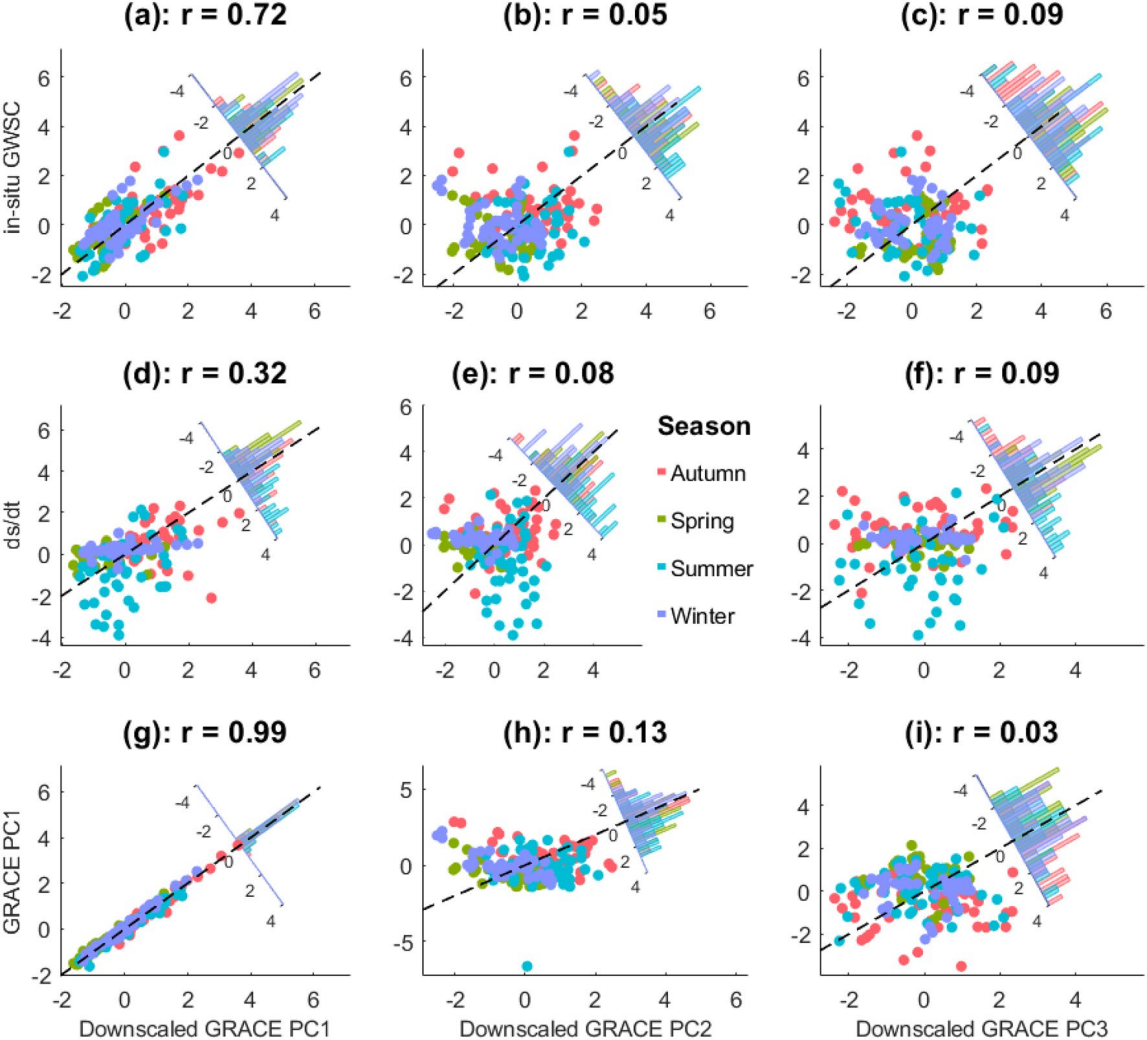
Scatterplots showing relationships of the downscaled GRACE PC’s with in-situ groundwater storage changes (**a**–**c**), water budget model (**d**–**f**) and the original GRACE PCs (**g**–**i**). Seasonal samples are shown with different coloured symbols. Spearman’s correlation coefficients are given for each plot. Also shown are frequency distributions of residuals from the line of perfect fit (indicated with dashed line). This plot was generated using MATLAB R2023a software—https://au.mathworks.com/products/matlab.html).

We further explored the consistency of the downscaled GRACE against in-situ ΔGWS and water budget estimates over different seasons and their performance using statistical methods (Table 4). The largest discrepancies were observed during the autumn and summer period due to the complex hydro-climatic activities during this normal wet season. During the autumn and summer season, temperature changes affect the state of water and influence ET rates, precipitation patterns are highly inconsistent, and the influence of natural and anthropogenic influences contributes to the complexity of this season, thus making it difficult to model than other seasons (Table 4). The idea for Fig. 6 was to examine the coherence of our downscaled products with the in-situ ΔGWS, ds/dt and the original GRACE under different hydrological conditions. While the autumn (March–May) and Winter (June–August) are characterized by significant latent heat transfers leading to high ET rates, low soil moisture, and a decline in the levels of surface waters, the spring months (September–November) and summer months (December to February) months are characterized by high humidity and possible cyclones (Fig. 3). These hydro-climatic events impact of the TWS over Australia and this was shown using our study area, however, we focus on the correlation between the downscaled products from PC1, PC2 and PC3 against in-situ GWS changes (Fig. 6a–c), water budget (Fig. 6d–f), and the original GRACE PC’s (Fig. 6g–i). PCA’s are very useful in identifying the dominant spatio-temporal patterns across seasons. For example, while the Northern part of Georgina basin witnessed a decline in TWS during the summer months, the southwestern part of the basin witnessed an increase in TWS during the same months (Figs. 5, 6).

**Table 4:** Results showing the performance metrics for (i) downscaled GRACE v. in-situ ΔGWS and (ii) downscaled GRACE v. ds/dt.

Season	Downscaled GRACE v. in-situ ΔGWS			Downscaled GRACE v. ds/dt		
	RMSE (mm)	NSE	MAE (mm)	RMSE (unitless)	NSE	MAE (unitless)
Autumn	100.6197	− 1.4196	0.9330	86.5958	− 0.7922	18.0223
Spring	46.2429	− 0.1946	0.4730	46.0947	− 0.1869	0.7840
Summer	60.2856	− 0.1089	0.5546	72.9322	− 0.6230	29.5537
Winter	58.6199	− 0.1472	0.3678	53.2491	0.0534	7.0870

We observed that the downscaled GRACE PC1, which contained most of the downscaled signals was significantly correlated with the in-situ GWS changes, the water budget, and the original GRACE estimates (Fig. 6a,d,g). This is because it contains a large chunk of the variance proportion compared to PC2 and PC3. This shows that PC2 and PC3 cannot be relied upon to depict the spatio-temporal changes of TWS over our study region and period. Figure 6a,d,g shows a significant spatio-temporal consistency between the PC1 (strongest variability PC) and the other products used for validation. The coherency across board makes it statistically significant. Therefore, we can safely report that our downscaled estimate can be relied upon for making significant estimates for other regions in Australia, where similar hydro-climatic conditions exist.

For a 0-month ahead lag time, our experiment shows a correlation coefficient of r = 0.70 between the downscaled GRACE and in-situ GWS changes, and r = 0.34 between the downscaled GRACE and the water budget (ds/dt) (Table 5). The hydrological flux variables of precipitation, ET and runoff were poorly correlated here at r = 0.06, 0.39 and 0.18, respectively. These values increased over the 1-month and 2-month lag times to accommodate the time it takes for hydrological fluxes to reflect on terrestrial water storage changes. The 2-month ahead lag times recorded the strongest relationship between the downscaled products and the water budget products while maintaining the smallest errors as shown in Table 5. This trend was also observed in the first (supporting information 8) PC plot, which is almost similar to the downscaled products due to its possession of ~ 90% signals, as well as the second (supporting information 9) and third PCs (supporting information 10) of the downscaled GRACE. This shows that the multi-annual signals are also sensitive to time lag changes of hydrological flux variables. We however, observed that the in-situ groundwater storage changes (GWSC) maintained the strongest relationship at the 0-month ahead lag time and the weakest correlation and the 2-month ahead lag time. This shows that the groundwater storage changes observed by the in-situ bores directly influence the observations from GRACE in real time. The strong correlations recorded between the downscaled GRACE and the water budget after lag adjustments shows that the downscaled GRACE is representative of the sub-grid heterogeneity and local-scale variations of water storage changes captured by the groundwater level variations over the CLA. On the other hand, the correlation between the downscaled GRACE and the water budget (even after lag adjustments) is at best average, which depicts that the inclusion of certain uncertainties in the water budget parameters makes land water storage understanding complex and is covered in the next section (5.2).

**Table 5:** Performance metrics of the downscaled GRACE signals against the water budget parameters and in-situ groundwater storage changes adjusted for 0, 1 and 2 months ahead lag times.

	Downscaled GRACE			
	r	RMSE (mm)	NSE	MAE (mm)
0-month ahead lag				
Precipitation	0.06	90.12	− 0.99	65.35
ET	0.39	61.75	0.09	48.55
Runoff	0.18	66.31	− 0.05	49.97
ds/dt	0.34	85.95	− 0.76	63.75
In-situ GWSC	0.70	54.85	0.28	41.31
1-month ahead lag				
Precipitation	0.47	69.22	− 0.14	52.14
ET	0.67	51.11	0.38	39.92
Runoff	0.50	61.80	0.09	46.14
ds/dt	0.02	76.45	− 0.39	56.10
In-situ GWSC	0.61	57.31	0.20	43.75
2-month ahead lag				
Precipitaion	0.63	59.17	0.17	44.43
ET	0.72	49.38	0.42	39.78
Runoff	0.51	61.69	0.10	45.74
ds/dt	0.26	69.13	− 0.13	50.08
In-situ GWSC	0.41	62.47	0.13	47.89

### Uncertainty assessment/limitations

#### AWO’s water budget

**Figure 7:**
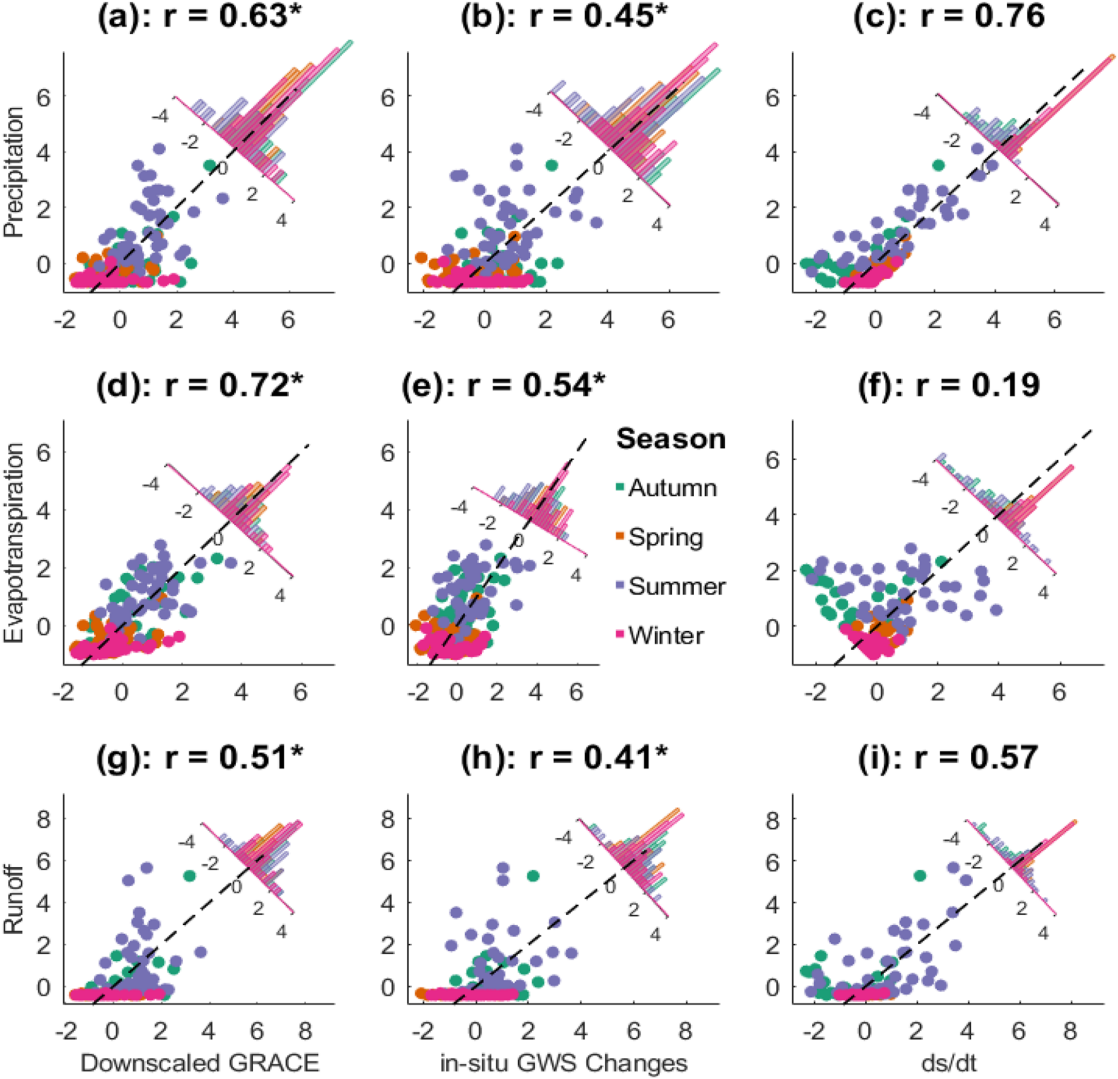
Correlation plot between the downscaled GRACE, in-situ GWS changes and the water balance model (ds/dt) against the hydrological fluxes of precipitation, evapotranspiration and runoff. The * in the regression values signifies parameters that were adjusted for a 2-month ahead time lag prior to their correlation. Seasonal samples are shown with different coloured symbols. Spearman’s correlation coefficients are given for each plot. Also shown are frequency distributions of residuals from the line of perfect fit (indicated with dashed line). This plot was generated using MATLAB R2023a software—https://au.mathworks.com/products/matlab.html).

The spatio-temporal variability evident in hydrological flux variables are driven by complex mechanisms ranging from climate variables and their interactions to anthropogenic influences. These are related to each other via the water budget equation. During our assessment of the water budget closure using the native AWO datasets (precipitation, ET, and runoff), it was observed that the ET values from the AWO are not only formed by the impacts of soil evaporation and vegetation transpiration, but also groundwater. Therefore, it becomes possible that these groundwater values present in the ET estimates may contribute to uncertainties in the AWO’s water budget closure for peculiar hydro-climatic regions in Australia. This is evident in Fig. 3h and l where the downscaled product did not match the water budget for the peak summer period. Given that the peak summer period is when ET is mostly dominant, its impact on our downscaling process is clearly based on its uncertainties. This was further confirmed in our study as we recorded a correlation of 0.20 between ET and the water budget model (ds/dt), while the precipitation and runoff fluxes correlated with the water budget at 0.76 and 0.57, respectively (Fig. 7c,f,i). Since ET can be said to be the most significant driver of the changes in our downscaled GRACE estimates when compared to other water budget terms (Fig. 7a,d,g), to improve our understanding of ET, we rely on the water budget equation. Previous studies have found that ET inferred from the water budget equation correlates with observational estimates, from either models or remote sensing platforms (in terms of seasonal cycles) but introduces larger magnitudes and larger inter-annual variabilities^27,68,69^, especially in summer months (Fig. 7f). More details can be found in supporting information 12.

#### In-situ Groundwater storage changes

A major limitation in this study is the uneven spread and sparse number of monitoring bores used in estimating in-situ groundwater storage changes over the study area (supporting information 11, Fig. 7b,e,h). This contributes to uncertainties in our experiments because accurate quantification of storage volume changes from in-situ observations is heavily reliant on an extensive monitoring bore network which was not available. Also, during the conversion of GWLs to GWSC, we adopted storage coefficient values from Knapton et al.^23^. The storage coefficients/specific yield estimates from Knapton et al.^23^ were developed using a 3D hydro stratigraphic block model which identifies lateral and vertical geological distributions having similar hydrogeological characteristics and then groups them in the same category. This model makes several assumptions based on the size of the basin and period of groundwater flows within the aquifer and is not designed to estimate storage coefficients in localized groundwater systems.

Our proposed downscaling method also contains uncertainties based on the interpolation of the missing months in the original GRACE datasets (JPL, CSR and GSFC), groundwater level readings, and the residuals obtained from the downscaling operation.

The uncertainty propagation of all the datasets used in our experiment is shown in supporting information 13.

### Advancing TWS downscaling using regional hydrological models

In the context of downscaling GRACE-TWS data using high-resolution hydrological fluxes, the idea is to utilize additional data sources, such as precipitation, ET, runoff, which are available at higher spatial resolutions. By incorporating these high-resolution hydrological datasets, it becomes possible to enhance the spatial details of the GRACE estimates and obtain more localized information about TWS changes.

Since our aim of using high resolution datasets is to obtain a better insight into the local hydrological processes of the region of interest, in this scenario, we argue that the use of estimates from regional hydrological models supersedes that from global hydrological models^56^. This is following the emergence of regional models as valuable tools for assessing and managing water resources at local scales^70^. While their global counterparts offer a broad understanding of the Earth’s hydrological system, regional models provide detailed insights into specific regions, enabling more accurate analysis of water availability, flood potential and the impacts of land use and climate change. By incorporating the uniqueness and high spatial resolution of the AWO model we were able to capture the effects of local precipitation patterns, evapotranspiration rates and runoff estimates in the water budget (Fig. 6a) which resulted in a more comprehensive understanding of the CLA’s changes in terrestrial water storage. The outputs from the AWO hydrological model provided spatially explicit information for our downscaling operation and improved the representation of the hydrological processes in the downscaled TWS estimates. Another useful aspect of regional models in the context of statistical downscaling lies in the inclusion of ancillary information, such as land cover details, soil properties, topography, and climate data, in accounting for the influence of TWS changes. Also, since estimates from regional models are often derived from ground-based observations and remote sensing products which have already been refined to capture local hydrological processes accurately, hydrological datasets from these models benefit from extensive calibration and validation efforts. By using these well-calibrated datasets, we can improve the accuracy and reliability of the downscaling process and enhance the confidence in the downscaled estimates. However, downscaling estimates can be improved with the introduction of additional predictor variables that represent and (or) contribute to the regional land water storage changes of specific regions, such as, soil moisture, surface water and even deep drainage estimates. This could result in the development of a more representative downscaled product which can be relied upon for local-scale water resource management and decision making.

## Conclusion

GRACE satellite has for the first-time enabled space-based detection of terrestrial water storage changes at large scales and in inaccessible regions. However, based on past water management policies, decision makers are usually more interested in water storage changes at finer scales than what GRACE offers. To meet this need and realize the full potential of the GRACE mission in hydrology, it is pertinent to improve the spatial resolution of GRACE data through downscaling. This study presents the use of support vector machine in downscaling GRACE data with high resolution predictors of precipitation, evapotranspiration, and runoff from the Australian Water Outlook model so that the final downscaled output is representative of local scale hydrological dynamics of the CLA.

Downscaling GRACE-TWS using high resolution precipitation, ET and runoff is an efficient way of identifying local-scale hydrological operations in relatively small catchments like the CLA. To validate our downscaled product, we used 12 in-situ groundwater monitoring stations spread unevenly across the study region. We also estimated trends from the water budget equation using the high-resolution predictors and performed statistical rotation using the principal component analysis on the original and downscaled products. These PC results from the downscaled TWS were compared to the in-situ groundwater level changes, water budget (ds/dt) and PC results from the original GRACE. With this operation, we were able to see that the downscaled PC1 products maintained a very high spatio-temporal consistency with the rest of the products, which was to be expected since it accounted for 90% of the total variability. The other PCs (i.e., PC2 and PC3) containing only strong intra-annual variations cannot be relied upon to depict water storage dynamics of the CLA. The downscaled PC1 also maintained good agreement with the validation products across the different Austral seasons which signifies that the downscaled product is useful and consistent with GRACE and can be replicated for other smaller regions within Australia. The major findings from this study are:

i. Statistical downscaling using regional hydrological models improves the ability of the downscaled product to characterize local-scale hydrological actions and represent small-scale features which may not be available in global hydrological models^56^.
ii. Machine learning applications in statistical downscaling of hydrological products are emerging as useful tools in analysing complex, local-scale hydrological systems/basins and predicating the availability, distribution, and dynamics of water resources in catchment scales.
iii. Complex hydrological basins like the CLA with inter-connected sub-basins having varied land water storage dynamics rely on regression-based downscaling operation to handle the non-linear relationships between the water budget estimates, surface and groundwater variables from each sub-basin. The capability of the machine learning regression models in quantifying the intricate relationships between these inter-connected water systems leads to an improved accuracy in predicting high-resolution downscaled details which are representative of the averaged local-scale hydrology of the catchment.

Our study also revealed that the possible uncertainties in the AWO’s evapotranspiration dataset could impact on downscaling. This is because, ET constitutes a major driver of TWS changes over our study region but maintained the least correlation with the land water storage changes of the region (ds/dt) when compared to other hydrological fluxes. The uncertainties are mostly evident in the summer months (December, January, February). The summer months are the hottest with the most significant latent heat transfers all year round and are characterized by irregular and sometimes intense rainfall events, leading to rapid changes in water storage. These high temperatures and prolonged sunlight during the summer months lead to significant evaporation rates, impacting water storage in lakes, rivers and reservoirs. Therefore, we recommend that future studies on the AWO’s ET dataset is critical for a more accurate assessment of terrestrial hydrology and by extension downscaling operations.

### Supplementary Information

Supplementary Information.

## Data Availability

High resolution predictor datasets used for our downscaling operation are freely available at: https://awo.bom.gov.au/products. GRACE-derived ΔTWS observations are available at https://podaac.jpl.nasa.gov/. In-situ groundwater levels datasets used for validation are available at http://www.bom.gov.au/water/groundwater/explorer/map.shtml.

## Code availability

We used MATLAB software for our statistical downscaling operation. The script is freely available for download from figshare via https://doi.org/10.6084/m9.figshare.25379125.v1.
