# Supplementary material for: Statistical downscaling of GRACE terrestrial water storage changes based on the Australian Water Outlook model
Source: Sci Rep. 2024 May 2;14:10113. doi: 10.1038/s41598-024-60366-2 (PMC11066110; doi:10.1038/s41598-024-60366-2)
Supplement: Supplementary file 1 — Supplementary Information. [file 41598_2024_60366_MOESM1_ESM.docx]

**Supplementary information for**

**Statistical downscaling of GRACE terrestrial water storage changes based on the Australian Water Outlook model**

Ikechukwu Kalu^a,b^, Christopher E. Ndehedehe^a,b^, Vagner G. Ferreira^c^ , Sreekanth Janardhanan^d^, Matthew Currell^e^ , Mark J. Kennard^a,b^

*^a^School of Environment & Science, Griffith University, Nathan, QLD 4111, Australia.*

*^b^Australian Rivers Institute, Griffith University, Nathan, QLD 4111, Australia.*

*^c^School of Earth Sciences and Engineering, Hohai University, Nanjing, China*

*^d^CSIRO Land and Water, Dutton Park, QLD 4102, Australia*

*^e^School of Engineering and Built Environment, Griffith University, Nathan, QLD 4111, Australia*

* Corresponding author.

Email address: [ikechukwu.kalu@griffithuni.edu.au](mailto:ikechukwu.kalu@griffithuni.edu.au) (Ikechukwu Kalu)


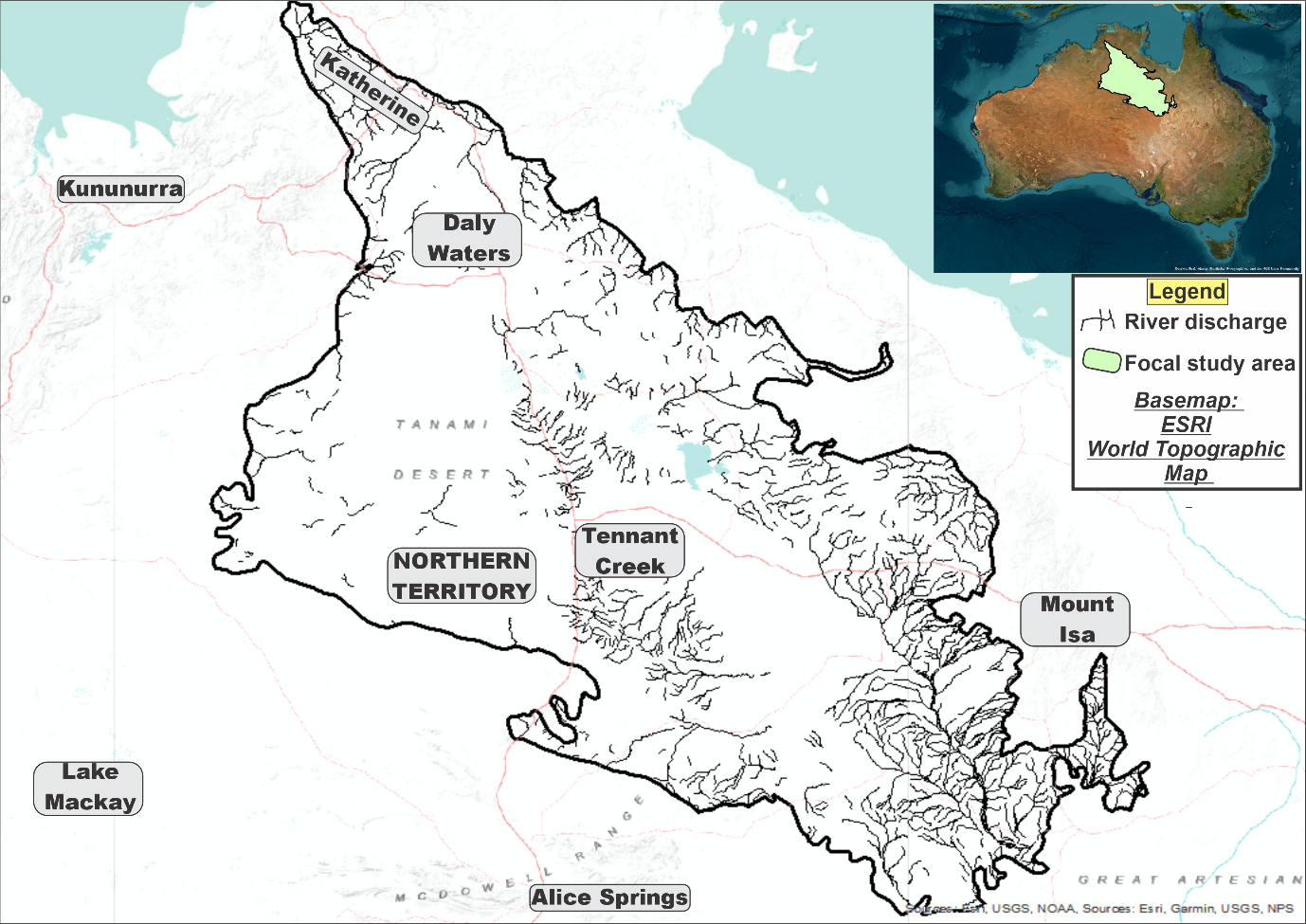


Supporting information 1a: Map showing the study region and spatial extent of our test bed (i.e. the Cambrian Limestone Aquifer). *Not drawn to scale.* This plot was generated using ArcMap 10.8 software - <https://www.esri.com/en-us/arcgis/products/arcgis-desktop/resources>)


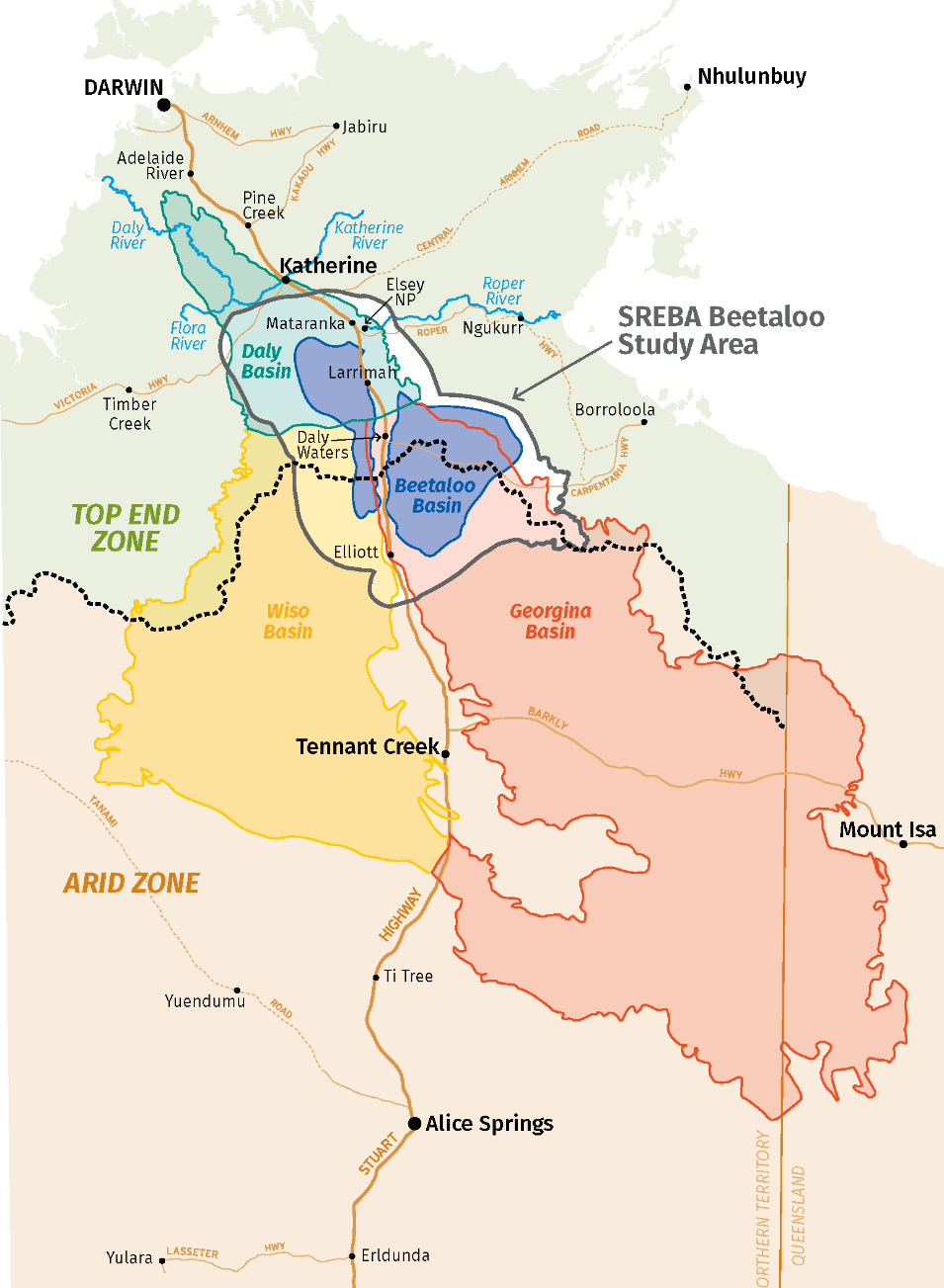


Supporting information 1b: The three sub-basins (Daly, Wiso and Georgina) of the CLA. *Not drawn to scale.* This plot was generated using ArcMap 10.8 software - <https://www.esri.com/en-us/arcgis/products/arcgis-desktop/resources>)

Supporting information 1c: Groundwater level data (in-situ)

Quality Description

A The record set is the best available given the technologies, techniques and monitoring objectives at the time of classification.

B The record set is compromised in its ability to truly represent the parameter.

C The record set is an estimate

E The record set’s ability to represent the monitored parameter is not known.

F The record’s set is not of release quality or contains missing data.

The readings were taken from depth to water level (DTW) estimates which are measured from the top of the ground surface to the groundwater level. This means that positive values are below the reference point and negative values are above the reference point.


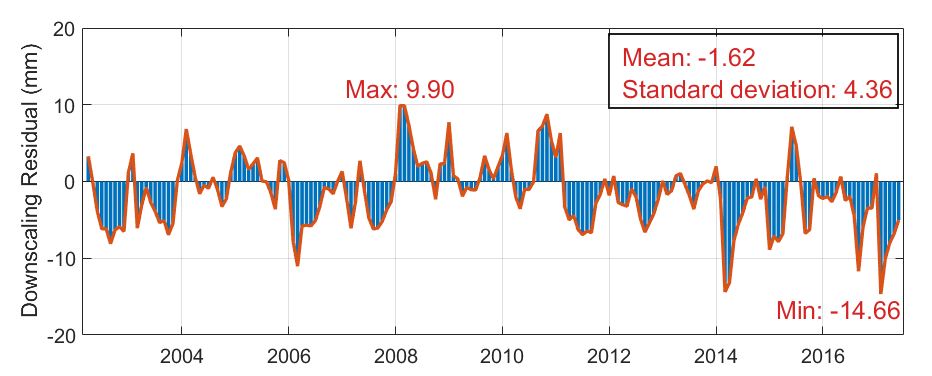


Supporting information 2: Time series of the residuals between the original and predicted GRACE TWS estimates of the support vector machine regression model. 2008 and 2017 recorded the strongest residual quantitates with max (9.90) and min (-14.66) values, respectively. These residuals account for the complex variabilities that may not be captured in the downscaling operation and must be added back to the predictions to correct systematic bias and ensure a more realistic representation of our downscaled products. This plot was generated using MATLAB R2023a software - <https://au.mathworks.com/products/matlab.html>)


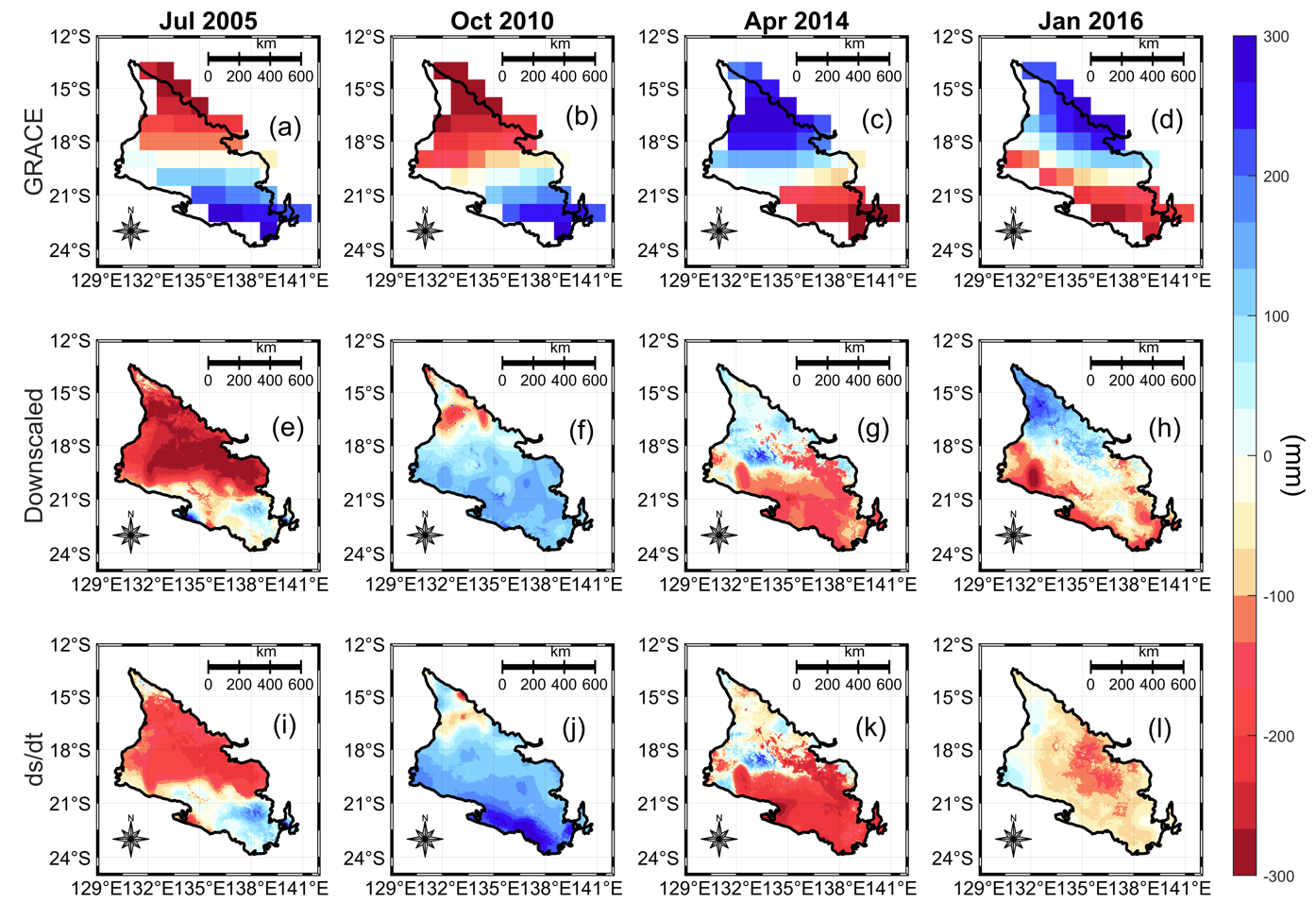


Supporting information 3: GRACE-CSR Terrestrial water storage changes over the CLA during the peak Austral winter (July), spring (October), autumn (April) and summer (January) seasons for selected years. This plot was generated using MATLAB R2023a software - <https://au.mathworks.com/products/matlab.html>)


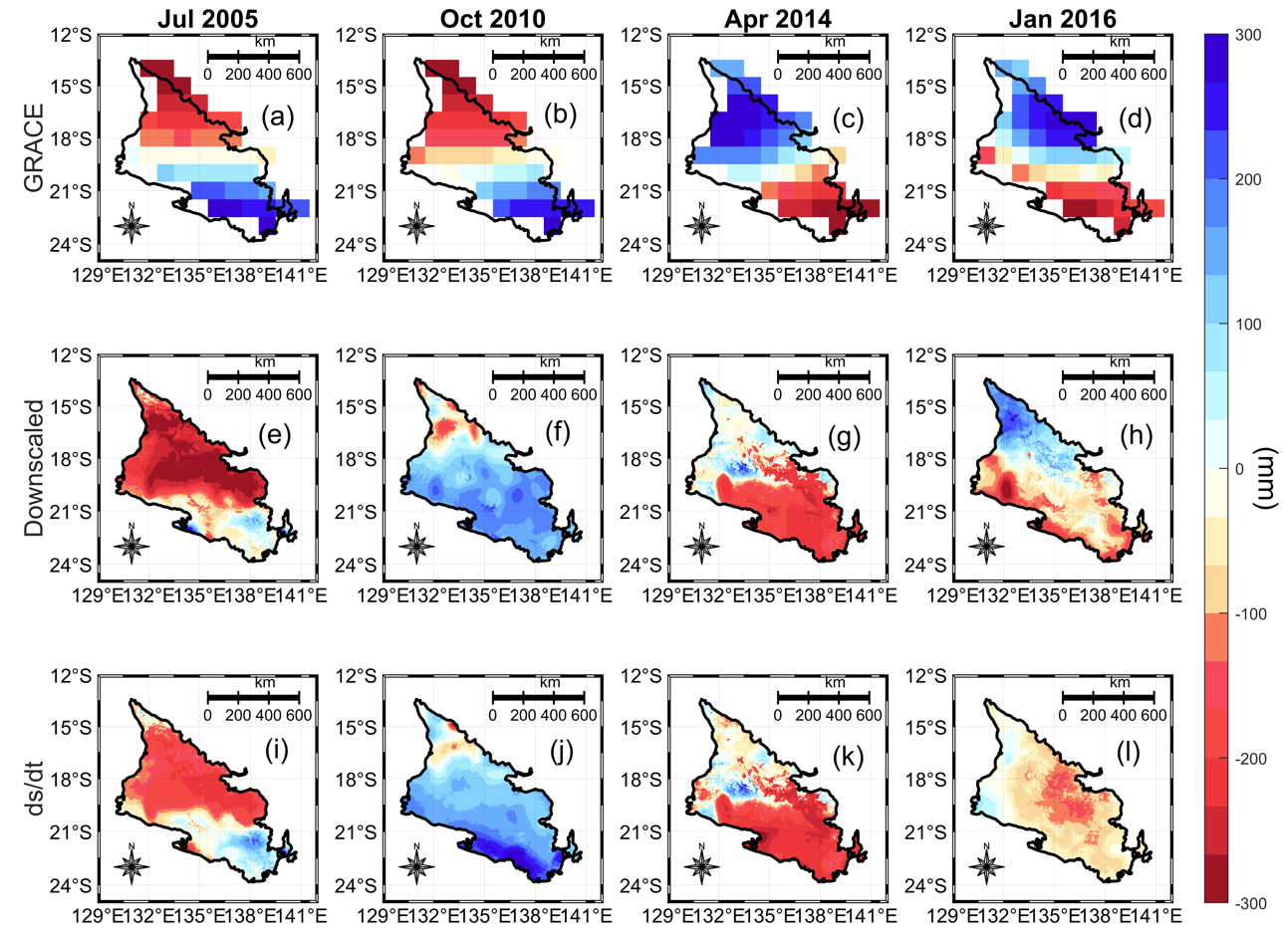


Supporting information 4: GRACE-JPL Terrestrial water storage changes over the CLA during the peak Austral winter (July), spring (October), autumn (April) and summer (January) seasons for selected years. This plot was generated using MATLAB R2023a software - <https://au.mathworks.com/products/matlab.html>)


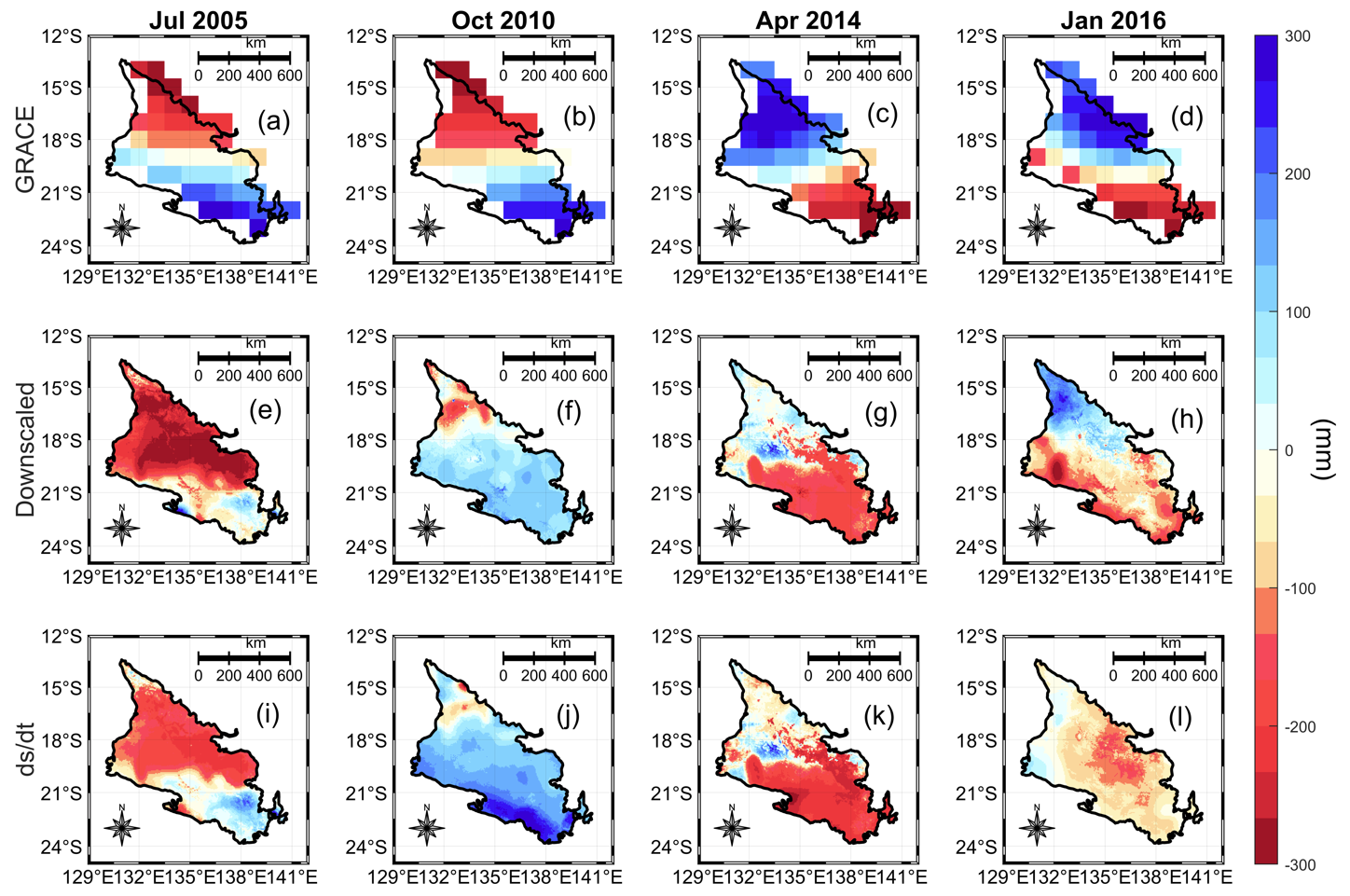


Supporting information 5: GRACE-GSFC Terrestrial water storage changes over the CLA during the peak Austral winter (July), spring (October), autumn (April) and summer (January) seasons for selected years. This plot was generated using MATLAB R2023a software - <https://au.mathworks.com/products/matlab.html>)


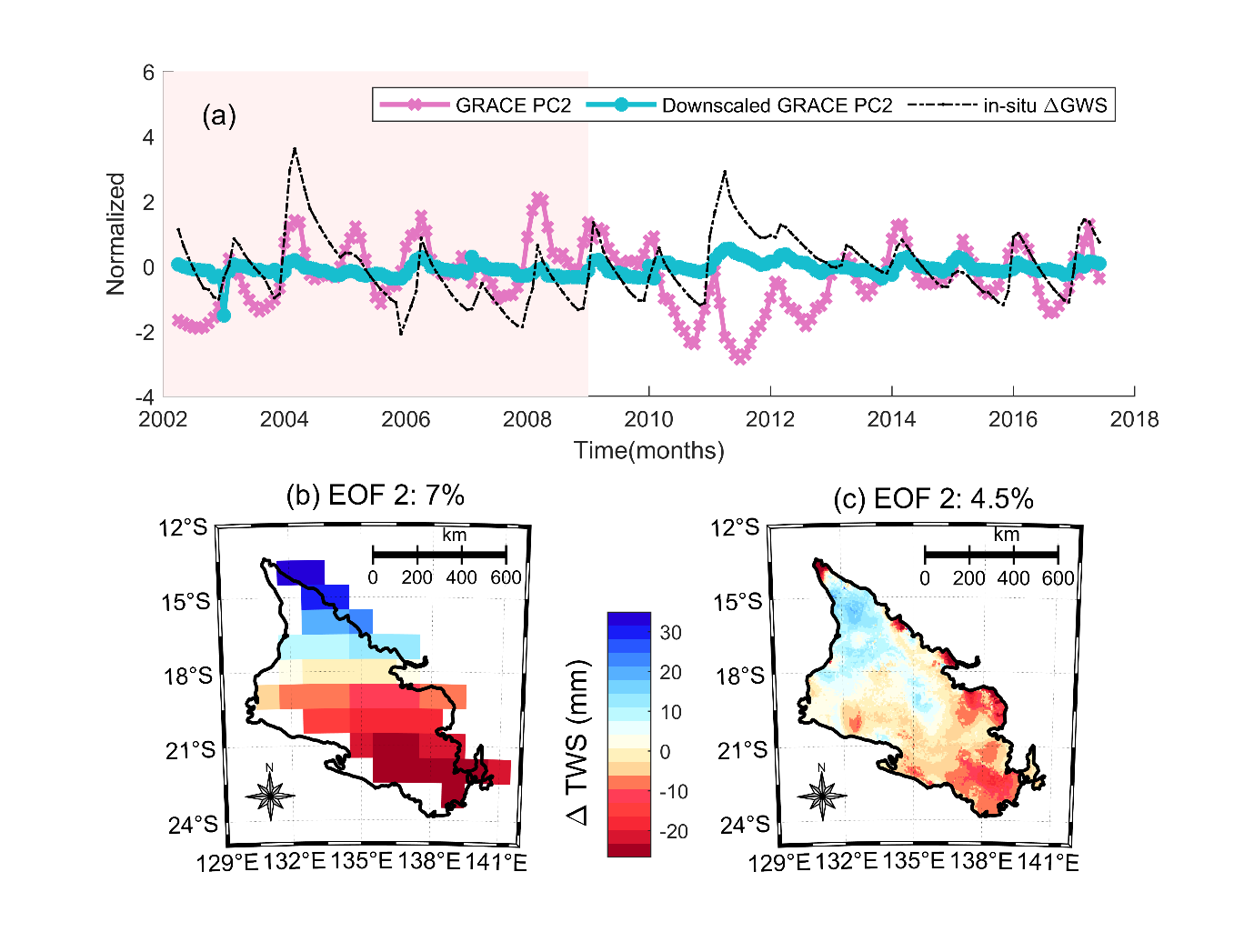


Supporting information 6: Validating our downscaled ΔTWS (PC 2) by checking its spatio-temporal consistency with the in-situ GWS changes and the original ΔTWS using principal component analysis. The pale red in (a) ranging from 2002 to 2009 represents the period of the Australia’s millennium drought which ended in 2009/2010. (b) and (c) depicts the empirical orthogonal functions (EOFs) of the original and downscaled GRACE, respectively. The EOFs are loadings showing spatial patterns of ΔTWS over the CLA while the corresponding PC2 (a) are temporal variations which are normalized using their standard deviations to be unitless. This plot was generated using MATLAB R2023a software - <https://au.mathworks.com/products/matlab.html>)


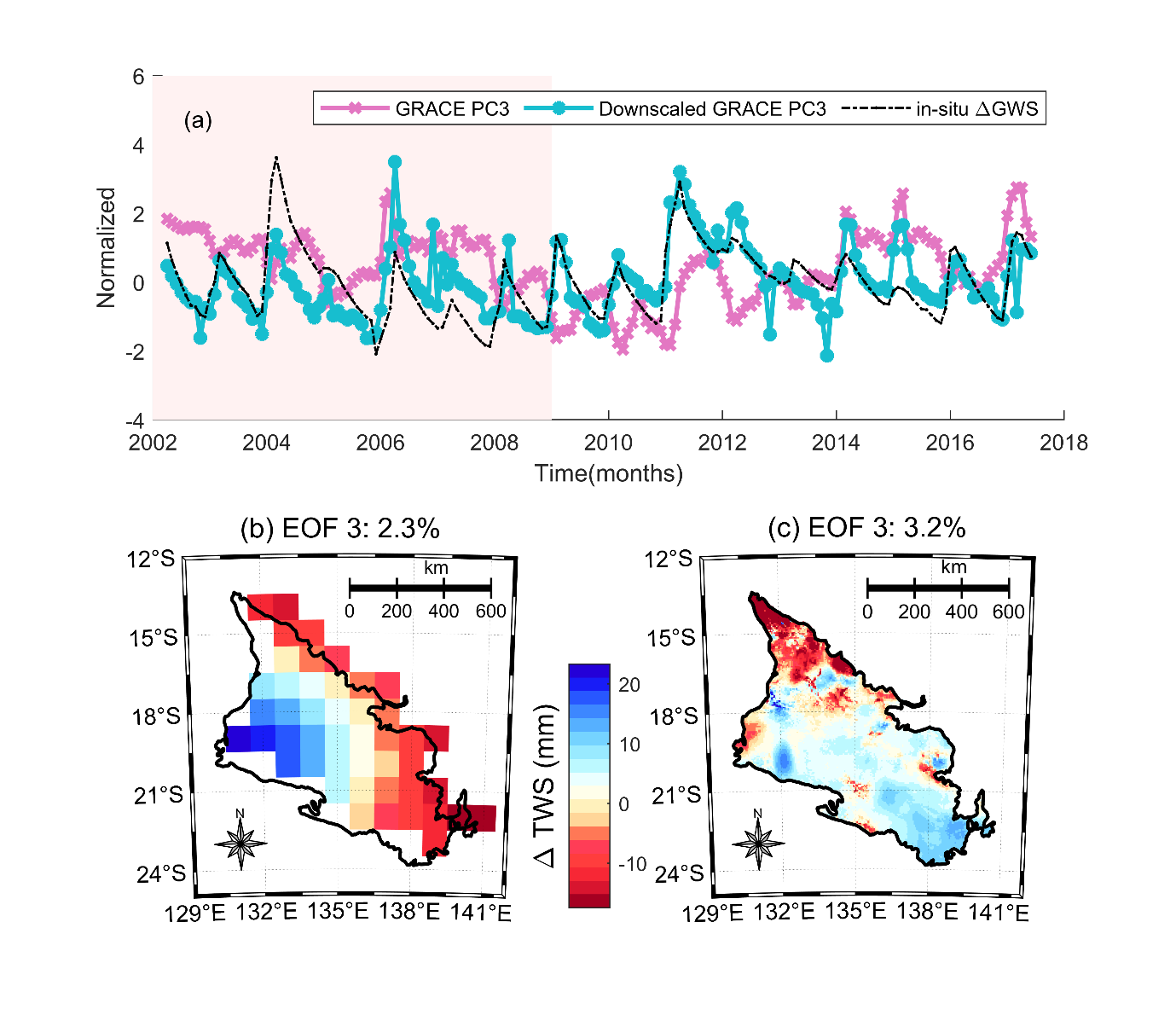


Supporting information 7: Validating our downscaled ΔTWS (PC 3) by checking its spatio-temporal consistency with the in-situ GWS changes and the original ΔTWS using principal component analysis. The pale red in (a) ranging from 2002 to 2009 represents the period of the Australia’s millennium drought which ended in 2009/2010. (b) and (c) depicts the empirical orthogonal functions (EOFs) of the original and downscaled GRACE, respectively. The EOFs are loadings showing spatial patterns of ΔTWS over the CLA while the corresponding PC3 (a) are temporal variations which are normalized using their standard deviations to be unitless. This plot was generated using MATLAB R2023a software - <https://au.mathworks.com/products/matlab.html>)

Supporting information 8: Performance metrics of the downscaled PC1 (EOF: 89.5%) signals against the water budget parameters and in-situ groundwater storage changes adjusted for 0,1 and 2 months ahead lag times.

| 0-month ahead lag | Downscaled GRACE PC1 | | | |
| --- | --- | --- | --- | --- |
|  | r | RMSE *(mm)* | NSE | MAE *(mm)* |
| Precipitation | 0.06 | 77.23 | -0.99 | 43.69 |
| ET | 0.39 | 50.01 | 0.09 | 37.70 |
| Runoff | 0.18 | 15.42 | -0.05 | 6.29 |
| ds/dt | 0.34 | 34.22 | -0.76 | 22.24 |
| in-situ GWSC | 0.70 | 22.70 | 0.28 | 17.61 |
| 1-month ahead lag | Downscaled GRACE PC1 | | | |
|  | r | RMSE *(mm)* | NSE | MAE *(mm)* |
| Precipitation | 0.47 | 77.11 | -0.14 | 43.87 |
| ET | 0.67 | 49.96 | 0.38 | 37.87 |
| Runoff | 0.50 | 15.16 | 0.09 | 6.19 |
| ds/dt | 0.02 | 33.96 | -0.39 | 22.04 |
| In-situ GWSC | 0.61 | 22.80 | 0.20 | 17.67 |
| 2-month ahead lag | Downscaled GRACE PC1 | | | |
|  | r | RMSE *(mm)* | NSE | MAE *(mm)* |
| Precipitaion | 0.63 | 77.19 | 0.17 | 44.08 |
| ET | 0.72 | 50.06 | 0.42 | 38.01 |
| Runoff | 0.51 | 15.18 | 0.10 | 6.17 |
| ds/dt | 0.27 | 33.79 | -0.13 | 21.86 |
| In-situ GWSC | 0.41 | 22.96 | 0.13 | 17.76 |

Supporting information 9: Performance metrics of the downscaled PC1 (EOF: 4.5%) signals against the water budget parameters and in-situ groundwater storage changes adjusted for 0,1 and 2 months ahead lag times.

| 0-month ahead lag | Downscaled GRACE PC2 | | | |
| --- | --- | --- | --- | --- |
|  | r | RMSE *(mm/mo)* | NSE | MAE *(mm/mo)* |
| Precipitation | 0.04 | 77.24 | -12.65 | 43.51 |
| ET | -0.27 | 50.25 | -5.56 | 37.63 |
| Runoff | -0.10 | 15.55 | -5.26 | 6.00 |
| ds/dt | 0.38 | 33.79 | -2.22 | 22.06 |
| in-situ GWSC | -0.58 | 23.47 | -7.86 | 18.22 |
| 1-month ahead lag | Downscaled GRACE PC2 | | | |
|  | r | RMSE *(mm/mo)* | NSE | MAE *(mm/mo)* |
| Precipitation | -0.39 | 77.52 | -12.71 | 43.76 |
| ET | -0.55 | 50.42 | -5.37 | 37.80 |
| Runoff | -0.41 | 15.65 | -5.18 | 6.07 |
| ds/dt | -0.02 | 33.96 | -2.44 | 22.20 |
| In-situ GWSC | -0.53 | 23.47 | -1.32 | 18.19 |
| 2-month ahead lag | Downscaled GRACE PC2 | | | |
|  | r | RMSE *(mm/mo)* | NSE | MAE *(mm/mo)* |
| Precipitaion | -0.51 | 77.76 | -12.71 | 44.01 |
| ET | -0.60 | 50.56 | -5.84 | 37.94 |
| Runoff | -0.44 | 15.70 | -5.50 | 6.12 |
| ds/dt | -0.19 | 34.09 | -2.01 | 22.30 |
| In-situ GWSC | 0.37 | 23.28 | -2.24 | 18.10 |

Supporting information 10: Performance metrics of the downscaled PC1 (EOF: 3.2%) signals against the water budget parameters and in-situ groundwater storage changes adjusted for 0,1 and 2 months ahead lag times.

| 0-month ahead lag | Downscaled GRACE PC3 | | | |
| --- | --- | --- | --- | --- |
|  | r | RMSE *(mm/mo)* | NSE | MAE *(mm/mo)* |
| Precipitation | -0.07 | 77.35 | -6.01 | 44.36 |
| ET | -0.35 | 50.50 | -2.56 | 37.70 |
| Runoff | -0.19 | 15.76 | -5.41 | 6.45 |
| ds/dt | 0.29 | 33.60 | -3.31 | 21.93 |
| in-situ GWSC | -0.64 | 24.00 | -2.45 | 18.63 |
| 1-month ahead lag | Downscaled GRACE PC3 | | | |
|  | r | RMSE *(mm/mo)* | NSE | MAE *(mm/mo)* |
| Precipitation | -0.44 | 77.86 | -15.22 | 43.96 |
| ET | -0.61 | 50.80 | -10.40 | 37.86 |
| Runoff | -0.45 | 16.03 | -9.97 | 6.58 |
| ds/dt | -0.05 | 34.02 | -9.25 | 22.32 |
| In-situ GWSC | -0.55 | 23.92 | -9.09 | 18.51 |
| 2-month ahead lag | Downscaled GRACE PC3 | | | |
|  | r | RMSE *(mm/mo)* | NSE | MAE *(mm/mo)* |
| Precipitaion | -0.54 | 78.16 | -18.89 | 44.25 |
| ET | -0.64 | 50.96 | -13.12 | 38.00 |
| Runoff | -0.45 | 16.08 | -10.44 | 6.64 |
| ds/dt | -0.20 | 34.26 | -10.01 | 22.55 |
| In-situ GWSC | -0.35 | 23.69 | -8.43 | 18.26 |


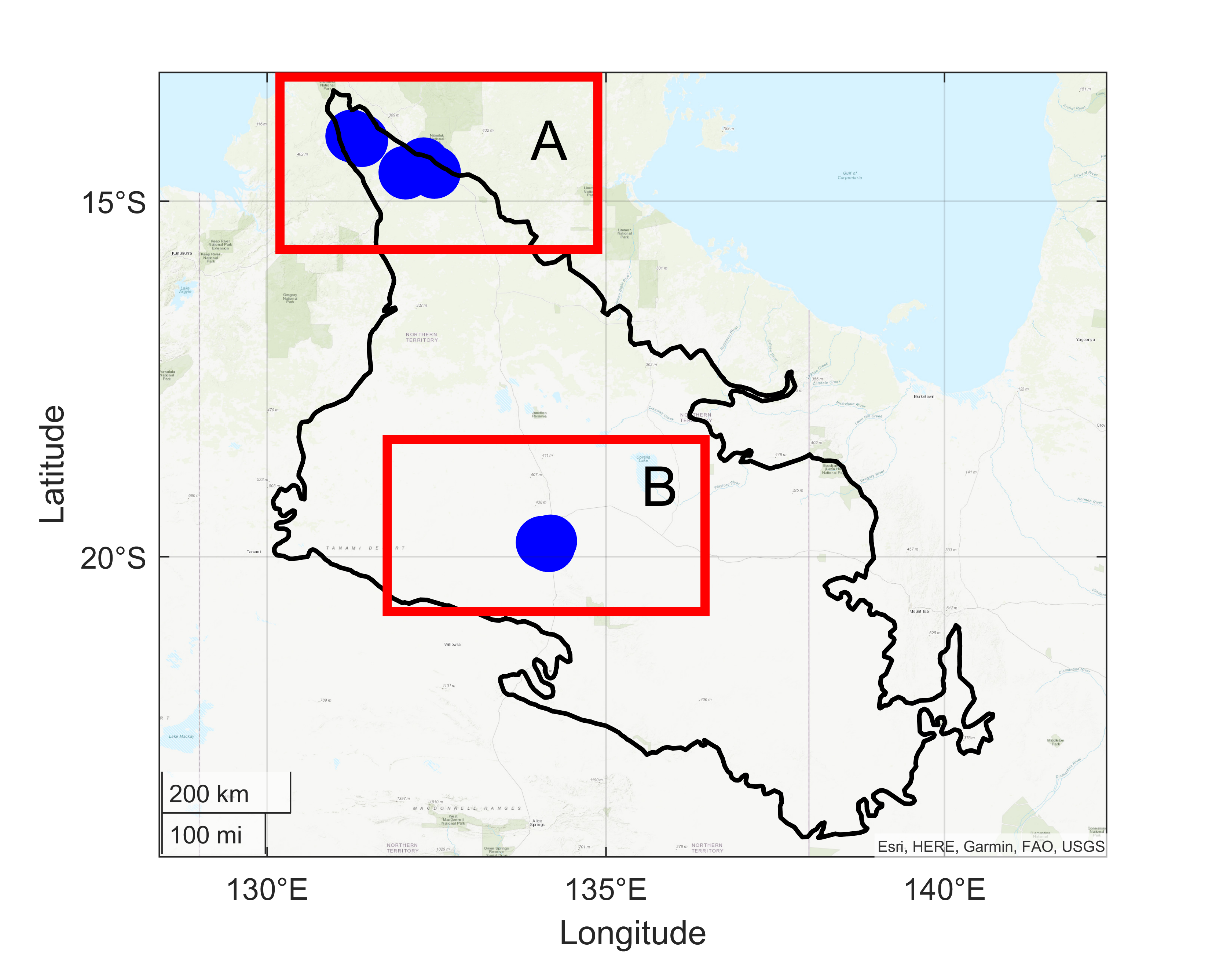


Supporting information 11: The uneven distribution of groundwater monitoring stations used in our experiment. There are 9 bores in box A and 3 bores in box B. The in-situ groundwater levels gotten from these networks of bores are averaged for the study period and used to estimate the groundwater storage changes of the CLA using equation 15. *Not drawn to scale.* This plot was generated using MATLAB R2023a software - <https://au.mathworks.com/products/matlab.html>)

Supporting information 12: Uncertainties in Evapotranspiration estimates

Evapotranspiration (ET) is a crucial component of the water cycle as it accounts for up to 60% of precipitation or even higher, in some regions [^1^](#_ENREF_1). It was also recorded as the most significant driver of the changes in our downscaled GRACE estimates when compared to the other hydrological flux parameters (Fig. 7a, d, g). [Rodell, et al. ^2^](#_ENREF_2) stated that ET constitutes the most significant uncertainty of the terrestrial water cycle, due to its high spatial variability and difficulties with direct measurement. The actual ET observation of the AWRA-L model estimates total evapotranspiration from vegetation, soil, and existing groundwater. The Strategic Regional Environmental and Baseline Assessment (SREBA) study [^3^](#_ENREF_3) found that most of the groundwater below the CLA (especially in the Georgina and Wiso basins) is very deep below the surface – e.g., >20m, which probably put the water below the maximum depth for effective ET. However, it remains likely that the ET values contain significant proportions of water drawn from groundwater due to groundwater flows from the Tindall limestone to the surface via springs and wetlands [^4^](#_ENREF_4). These flows, in the form of groundwater discharge also support permanent vegetation, aquatic ecosystems within spring pools, groundwater dependent sections of streams and downstream water bodies which all contribute to ET rates over the region [^4^](#_ENREF_4). This shows that these ET values might consists of more uncertainties, and this should be noted before being applied in water budget studies and hydrological analysis. Though the AWRA-L report states that the evaporation from groundwater occurs only in saturated parts of the landscape where the water table intersects on the surface [^5^](#_ENREF_5), we cannot rule out the effects of groundwater on soil moisture in regions with shallow groundwater table. In fact, [Chen and Hu ^6^](#_ENREF_6) suggested that groundwater can act as a soil water source and have substantial effects in areas where the water table is near or within a model’s soil column. Due to the groundwater effects on soil moisture and the local terrestrial water cycle, spatial variations in groundwater table depth could lead to spatial heterogeneity in soil moisture and, afterward, surface moisture flux across the region. The significance of such heterogeneity plays a major role in the regional evaporation of any space and can influence the water budget in ways not captured by the models.

Besides unconfined aquifers, shallow groundwater tables affect soil moisture and contributes to uncertainties in ET estimation. Unfortunately, past studies of water exchange between the unsaturated zone and the atmosphere attempted to explore soil moisture variations and their effects on atmospheric boundary layer processes affecting climate but still neglect groundwater effects on soil moisture [^6-8^](#_ENREF_6). The water budget equation for a closed system proposes an ideal situation where the ET values are directly estimated quantities of precipitation and nothing else. Therefore, a case where a steady source of uncertainty such as the effects of underlying groundwater depth on the ET estimates remains unresolved, will result in an unbalanced water budget equation in peculiar regions.

Supporting information 13: Uncertainty propagation of all the datasets used in this experiment. Their absolute uncertainties are estimated at a 95% confidence interval with a t-value of 1.9731 (two-tailed test).

| Variable | Average | Standard deviation | Absolute uncertainty (mm) | % Uncertainty |
| --- | --- | --- | --- | --- |
| Precipitation | 43.5630 | 64.0089 | 9.3361 | 21.43% |
| Evapotranspiration | 37.7045 | 33.3277 | 4.8610 | 12.89% |
| Runoff | 5.9741 | 14.3985 | 2.1001 | 35.15% |
| ΔTWS (CSR) | 22.5653 | 63.9809 | 9.3320 | 41.36% |
| ΔTWS (JPL) | 23.7607 | 63.2712 | 9.2285 | 38.84% |
| ΔTWS (GSFC) | 26.5123 | 62.8433 | 9.1661 | 34.57% |
| ΔTWS (Mean) | 24.2794 | 63.0902 | 9.2021 | 37.90% |
| MB 1: RN005248 | 12.5606 | 0.2524 | 0.0368 | 0.29% |
| MB 2: RN010167 | 8.2816 | 0.9904 | 0.1445 | 1.75% |
| MB 3: RN010564 | 14.8195 | 0.9720 | 0.1418 | 0.96% |
| MB 4: RN033033 | 15.8882 | 2.0088 | 0.2930 | 1.83% |
| MB 5: RN002522 | 6.2278 | 2.0989 | 0.3061 | 4.94% |
| MB 6: RN034595 | 15.0542 | 1.5057 | 0.2196 | 1.46% |
| MB 7: RN022394 | 7.8947 | 2.0812 | 0.3036 | 3.84% |
| MB 8: RN034364 | 8.0318 | 1.4575 | 0.2126 | 2.63% |
| MB 9: RN029429 | 1.4852 | 1.1852 | 0.1729 | 11.51% |
| MB 10: RN034597 | 11.7210 | 1.4329 | 0.2090 | 1.79% |
| MB 11: RN008221 | 20.4638 | 2.3745 | 0.3463 | 1.69% |
| MB 12: RN034596 | 11.7003 | 1.4789 | 0.2157 | 1.85% |
| MB (Mean) | 10.9917 | 0.9990 | 0.1457 | 1.33% |

*MB represents Monitoring Bores,* degree of freedom = Time in months – 1, Standard deviation represents the water level measurements around the mean value.

The average and standard deviation estimates of these datasets are vital in the uncertainty propagation of the the water budget terms, monitoring bores and GRACE datasets used in our experiments. Absolute and percentage uncertainty represents the magnitude of the potential error in the measurement being assessed and were computed as

$abs_{unc}=tv \times\frac{SD}{\sqrt{T}}$ (S1)

where $abs_{unc}$ is the absolute uncertainty, tv is the t-value computed at a 95% confidence interval, SD is the standard deviation of the datasets and T represents the time in months.

$\%U= \left( \frac{abs_{unc}}{\bar{X}} \right) \times100$ (S2)

where %U is the percentage uncertainty and $\bar{X}$ represents the mean/average of the dataset.

**References**

1 Oki, T. & Kanae, S. Global Hydrological Cycles and World Water Resources. *Science* **313**, 1068-1072 (2006). <https://doi.org/doi:10.1126/science.1128845>

2 Rodell, M. *et al.* The Observed State of the Water Cycle in the Early Twenty-First Century. *Journal of Climate* **28**, 8289-8318 (2015). <https://doi.org/https://doi.org/10.1175/JCLI-D-14-00555.1>

3 Department of Environment, P. a. W. S. Regional Report: Strategic Regional Environmental and Baseline Assessment for the Beetaloo Sub-basin. Report No. 41/2022, (Department of Environment, Parks and Water Security, Northern Territory Government. Berrimah, Northern Territory. , 2022).

4 Currell, M. & Ndehedehe, C. E. The Cambrian Limestone Aquifer, Northern territory: Review of the hydrogeology and management rules to ensure protection of groundwater dependent values. (2022).

5 Frost, A. J. & Shokri, A. The Australian Landscape Water Balance model (AWRA-L v7). Technical Description of the Australian Water Resources Assessment Landscape model version 7. (2021).

6 Chen, X. & Hu, Q. Groundwater influences on soil moisture and surface evaporation. *Journal of Hydrology* **297**, 285-300 (2004). <https://doi.org/https://doi.org/10.1016/j.jhydrol.2004.04.019>

7 Pielke, R. A. & Avissar, R. Influence of landscape structure on local and regional climate. *Landscape Ecology* **4**, 133-155 (1990). <https://doi.org/10.1007/BF00132857>

8 Pielke, R. A., Dalu, G. A., Snook, J. S., Lee, T. J. & Kittel, T. G. F. Nonlinear Influence of Mesoscale Land Use on Weather and Climate. *Journal of Climate* **4**, 1053-1069 (1991). <https://doi.org/https://doi.org/10.1175/1520-0442(1991)004><1053:NIOMLU>2.0.CO;2
